# Seasonal Niche Partitioning of Surface Temperate Open Ocean Prokaryotic Communities

**DOI:** 10.3389/fmicb.2020.01749

**Published:** 2020-07-28

**Authors:** Catalina Mena, Patricia Reglero, Rosa Balbín, Melissa Martín, Rocío Santiago, Eva Sintes

**Affiliations:** Instituto Español de Oceanografía (IEO), Centre Oceanogràfic de les Balears, Ecosystem Oceanography Group (GRECO), Palma, Spain

**Keywords:** microbial communities, marine prokaryotes, oligotyping, seasonality, 16S ribosomal RNA gene, open ocean, Mediterranean Sea, association networks

## Abstract

Surface microbial communities are exposed to seasonally changing environmental conditions, resulting in recurring patterns of community composition. However, knowledge on temporal dynamics of open ocean microbial communities remains scarce. Seasonal patterns and associations of taxa and oligotypes from surface and chlorophyll maximum layers in the western Mediterranean Sea were studied over a 2-year period. Summer stratification versus winter mixing governed not only the prokaryotic community composition and diversity but also the temporal dynamics and co-occurrence association networks of oligotypes. Flavobacteriales, Rhodobacterales, SAR11, SAR86, and Synechococcales oligotypes exhibited contrasting seasonal dynamics, and consequently, specific microbial assemblages and potential inter-oligotype connections characterized the different seasons. In addition, oligotypes composition and dynamics differed between surface and deep chlorophyll maximum (DCM) prokaryotic communities, indicating depth-related environmental gradients as a major factor affecting association networks between closely related taxa. Taken together, the seasonal and depth specialization of oligotypes suggest temporal dynamics of community composition and metabolism, influencing ecosystem function and global biogeochemical cycles. Moreover, our results indicate highly specific associations between microbes, pointing to keystone ecotypes and fine-tuning of the microbes realized niche.

## Introduction

Marine microbes are crucial players of ocean element cycles and modulate global biogeochemical processes (Azam and Malfatti, 2007; Fuhrman, 2009; Moran, 2015). Autotrophic prokaryotes account for 8–19% of primary production in the sunlit oligotrophic ocean regions (Rousseaux and Gregg, 2013), contributing significantly to the export of carbon and energy that fuels the dark ocean communities (Johnson and Lin, 2009; Guidi et al., 2016). Prokaryotic communities transfer energy to other trophic levels through grazing (Pernthaler, 2005; Vaque et al., 2014), viral lysis (Suttle, 2007; Chen et al., 2019), and organic matter decomposition and remineralization (Kujawinski, 2011; Buchan et al., 2014).

Marine surface microbial communities show remarkable seasonal dynamics, driven by environmental seasonality patterns (Fuhrman et al., 2015; Bunse and Pinhassi, 2017). Temperature, light intensity, nutrient availability, water mixing, and stratification are seasonally related factors that regulate microbial community dynamics (Morris et al., 2005; Malmstrom et al., 2010; Gilbert et al., 2012; Sintes et al., 2013; Pearman et al., 2017), originating recurrent patterns in community composition and functionality (Giovannoni and Vergin, 2012; Teeling et al., 2012; Fuhrman et al., 2015). Growth rates and activity of specific prokaryotic taxa are also strongly influenced by seasonal fluctuations (Alonso-Sáez and Gasol, 2007; Hugoni et al., 2013).

Additionally, community structure is influenced by microbial interactions (Faust and Raes, 2012). A plethora of organism interactions have been evidenced that alter community metabolism and function (Azam and Malfatti, 2007; Fuhrman, 2009; Teira et al., 2019). Interactions can result in positive, negative, or neutral impact on the organisms involved, including mutualism, commensalism, parasitism or predation, and amensalism (Faust and Raes, 2012). Quorum sensing is a microbe interaction mechanism through chemical communication involved in different processes of marine biogeochemical cycles, trophic interactions and biofilm formation and functioning (Hmelo, 2017). The release of extracellular products or metabolites derived from microorganism’s metabolism may also result in microbe-microbe interactions (Li et al., 2015; Reintjes et al., 2019). Free amino acids and other organic compounds released by phytoplankton and zooplankton are a significant energy and carbon source for marine prokaryotes (Williams et al., 2013; Buchan et al., 2014; Clifford et al., 2019). Alteromonadaceae release exo-enzymes to hydrolyze complex compounds into simpler molecules, which can potentially be assimilated by other microbes such as SAR11 (Reintjes et al., 2019). Symbiosis between cyanobacteria and eukaryotic algae has also been reported (Martínez-Pérez et al., 2016). Interactions may also be negative for one or both microbes involved, e.g., competition for limiting resources (Teira et al., 2019), predation (Pernthaler, 2005; Banning et al., 2010) or release of inhibitory compounds (Long and Azam, 2001). Alteromonadales and Vibrionales, either free-living or surface-attached, produce and release inhibitory compounds, strongly reducing Bacteroidetes growth (Long and Azam, 2001). However, interactions between microbes are challenging to resolve.

Correlation-based network-analysis (Liu et al., 2019) is receiving increased attention as a tool to advance our knowledge of connectivity between microbial taxa. Studies based on operational taxonomic units (OTUs) have revealed seasonal cycles and co-occurrence patterns among ammonia-oxidizing archaea, SAR11, SAR86, Cyanobacteria, Actinobacteria, and Flavobacteria microbial OTUs and other common groups (Beman et al., 2011; Chow et al., 2013), outlining potential ecological networks. Nevertheless, the association (positive or negative) between microbes can be caused by the interplay between organisms but also by a similar response of these organisms to the environmental conditions, not involving direct microbe interactions.

In addition, the ecological significance of taxa, specially at high taxonomic ranks, is still debated (Philippot et al., 2010). A recent approach developed to resolve closely related groups of ecological significance is oligotyping analysis (Eren et al., 2013). This analysis identifies varying positions in nucleotide (Eren et al., 2013) or protein (Sintes et al., 2016) sequences of specific taxonomic groups, generating oligotypes and revealing different ecological patterns within the specific taxon (i.e., ecotypes). Seasonal recurring dynamics have been observed between SAR11 ecotypes, related to winter physical mixing and stratification of the water column (Treusch et al., 2009; Vergin et al., 2013a; Salter et al., 2015). Abundant and rare archaeal ecotypes have also shown seasonal patterns, exhibiting temporal fluctuations in abundance and activity (Hugoni et al., 2013). Seasonal changes in light, temperature, and water mixing drive temporal patterns of depth-differentiated *Prochlorococcus* ecotypes (Malmstrom et al., 2010). Information on temporal dynamics of ecotypes is essential to determine ecological niches and fine-scale temporal community dynamics, extending our knowledge on microbial interactions and ecosystem functioning. However, studies on ecotype seasonality, specially of open ocean communities, are still scarce.

The aims of the study were to (i) further characterize seasonal and spatial dynamics of prokaryotic communities and oligotypes of abundant taxa from surface and chlorophyll maximum layers in the open western Mediterranean Sea and (ii) assess inter-oligotype connections. Oligotyping analysis was performed on 16S ribosomal RNA (rRNA) gene sequences and correlation-based networks were obtained. The results provide new insights into the temporal niche partitioning and associations of prokaryotic phylotypes.

## Materials and Methods

### Study Site and Sampling

The sampling was carried out seasonally from February 2016 to December 2017 at five stations in the western Mediterranean Sea during the RADMED cruises. Campaigns in February, April, July, and October 2016 and February, June, and November 2017 were, hereinafter, named Feb16, Apr16, Jul16, Oct16, Feb17, Jun17, and Nov17, respectively. Campaigns were grouped as follows, in order to discuss the seasonal patterns: Feb16 and Feb17 campaigns are referred to as winter, Apr16 as spring, Jul16 and Jun17 as summer, and Oct16 and Nov17 campaigns as autumn. Specific sampling dates for each station are detailed in Supplementary Table S1. The stations were located in different regions of the basin: north Balearic sub-basin (A), Mallorca channel (B), south Balearic sub-basin (C), north Algerian sub-basin (D), and south Algerian sub-basin (E) (Supplementary Figure S1).

Seawater from the upper 200 m was collected using Niskin bottles mounted on a SBE911 conductivity-temperature-depth (CTD) rosette sampler equipped with oxygen (SBE43), Chlorophyll-*a* (Chl-*a*) fluorescence (SeaPoint Fluorometer) and photosynthetically active radiance (PAR and SPAR Biospherical) sensors. The thermocline was defined as the layer at the base of the surface mixed layer in which the temperature vertical gradient was more pronounced. Water samples for inorganic nutrients, Chl-*a*, picophytoplankton abundances, and total prokaryotic abundance were collected at 0, 25, 50, 75, 100, and 200 m and at the deep chlorophyll maximum (DCM) depth. The prokaryotic community composition was assessed at 0 m and DCM depth.

### Inorganic Nutrients and Chlorophyll-*a*

Twelve milliliter of seawater for dissolved inorganic nutrients was collected and stored frozen at −20°C until further analysis at the home lab. Nitrate (NO_3_^−^), nitrite (NO_2_^−^), phosphate (PO_4_^3−^), and silicate (SiO_4_^2−^) concentrations were analyzed with a QuAAtro gas segmented continuous flow analyzer (SEAL analytical) following standard methods (Murphy and Riley, 1962; Strickland and Parsons, 1968; Grasshoff et al., 1983). Detection limits were 0.023, 0.01, 0.007, and 0.030 μM for nitrate + nitrite, nitrite, phosphate, and silicate, respectively.

Chl-*a* concentration was measured spectrophotometrically after filtering 1 L of seawater onto GF/F Whatman glass fiber filters. Chl-*a* from microbes retained on the filters was extracted in cold acetone (90%) for 24 h and analyzed with a turner-designs 10AU fluorometer. Phaeopigments concentration was analyzed after adding two drops of 1.2 N HCl. The measurements prior to and after acidification were used to calculate concentration of Chl-*a* and phaeopigments, respectively (Holm-Hansen et al., 1965; Lorenzen, 1967).

### Picophytoplankton and Total Prokaryotic Abundances

Microbial abundances were measured by flow cytometry. Seawater (1.5 ml) was fixed with glutaraldehyde (0.1% final concentration) for 10 min, frozen in liquid nitrogen and stored at −80°C until further analysis. Prior to enumeration, fluorescent beads (Fluospheres polystyrene 1.0 μm, Molecular probes) were added to all samples as internal standard.

Picophytoplankton groups were counted on a FACSAria II flow cytometer (BD Biosciences). *Prochlorococcus*, *Synechococcus*, and picoeukaryotes were differentiated based on their side scatter versus red fluorescence and orange fluorescence versus red fluorescence signals (Marie et al., 2000). Cytogram plots were used to draw a gate for each group with the BD FACSDiva software. Flow rate was measured daily.

Total prokaryotes were enumerated on an ACCURI C6 flow cytometer (BD Biosciences), after staining with SYBR Green I (Sigma-Aldrich, 1× final concentration) for 10 min in the dark. High nucleic acid (HNA) and low nucleic acid (LNA) content cells were separated based on their side scatter versus green fluorescence signals (Brussaard, 2004). Gating on cytogram plots was adjusted for each sample.

### Prokaryotic Community Composition

Four liters of seawater were filtered onto polycarbonate filters (0.2 μm pore size, 47 mm diameter, Whatman, Nucleopore), immediately frozen in liquid nitrogen and stored at −80°C. At the lab, filters were thawed, cut into small pieces and incubated 45 min at 37°C with lysis buffer containing lysozyme. Subsequently, filter pieces were incubated 1 h at 55°C with proteinase K. Zirconium beads were added and the lysate was subjected to beat-beating for 10 min, followed by 30 min incubation at 70°C. Subsequently, DNA was extracted successively with phenol (pH8), phenol-chloroform-isoamylalcohol (25:24:1), and chloroform. DNA was precipitated with two volumes of cold ethanol and 0.02 volumes of 5 M NaCl at −20°C overnight. DNA was pelleted by centrifugation (25 min, 21,000 × *g*), washed with cold ethanol (70°C), resuspended in sterile DNAse-RNAse free water and stored frozen at −80°C until further analysis.

16S rRNA gene of Archaea and Bacteria was amplified using primers 515F-Y (5'-GTGYCAGCMGCCGCGGTAA) and 926R (5'-CCGYCAATTYMTTTRAGTTT) (Parada et al., 2016). PCR cycling was performed following Parada et al. (2016). PCR products were purified using the Qiaquick PCR purification kit (Qiagen, Hilden) according to manufacturer’s instructions, and checked on a 2% agarose gel. Sequencing was performed with Illumina MiSeq 2 × 250 bp and analyzed using QIIME2^1^. Quality check, denoise, and dereplication of paired-end sequence reads were performed using DADA2 algorithm (Callahan et al., 2016) implemented in QIIME2. Sequences with ambiguities were removed (maxN = 0) and the length of forward and reverse reads was truncated to 245 and 230 bp, respectively. After filtering, a total number of 547,992 sequences were retained for further analyses, ranging from 4,707 to 13,709 sequences per sample. The obtained amplicon sequence variants (ASVs) were aligned with multiple alignment using fast Fourier transform (MAFFT) (Katoh and Standley, 2013) and a phylogenetic tree was built with Fasttree (Price et al., 2010). The ASVs table, rarefied to 4,707 sequences, was used to calculate Shannon’s diversity, Pielou’s evenness, phylogenetic diversity indexes, and unweighted UniFrac distances. Taxonomic assignment was obtained based on the SILVA database (release 132). 16S rRNA raw sequences were deposited in the NCBI database, accession number PRJNA612168.

### Oligotyping

Oligotyping analysis was performed on the most abundant phylotypes at the order level (>3% relative abundance in the photic layer), including main representatives of the phyla/classes of both Archaea and Bacteria: SAR11, Synechococcales, Chloroplast, Flavobacteriales, Marine Group II, Rhodobacterales, SAR86, Actinomarinales, and Alteromonadales, following the pipeline described in http://oligotyping.org. Oligotyping identifies closely related taxa consistently differing in nucleotide composition at specific positions (Eren et al., 2013), helping resolve ecologically significant populations. We complemented ASVs with oligotyping analysis in order to group ASVs based on an information-theoretic approach instead of arbitrary sequence identity thresholds and to infer ecologically meaningful groups (i.e., ecotypes). Between 7 and 14 entropy positions were used to assess the oligotypes of the abovementioned phylotypes (see Table 1). Oligotypes that occurred in more than 0.5% of reads (*a* = 0.5) were considered.

**Table 1:** Oligotyping results for the nine phylotypes analyzed at the order level.

Phylotypes	Total reads	ASVs	Taxa	Entropy positions	Oligotypes
SAR11	101,648	583	20	14	22
Synechococcales	38,073	32	7	8	29
Chloroplast	43,332	559	30	7	67
Flavobacteriales	82,012	1,023	73	7	103
Marine Group II	17,254	174	11	9	26
Rhodobacterales	21,336	425	14	7	47
SAR86	25,101	457	7	8	26
Actinomarinales	18,876	122	7	7	44
Alteromonadales	25,734	336	14	8	33

Total number of reads, number of ASVs assigned to each phylotype, number of taxa assigned to each order according to SILVA classifier (release 132), number of entropy positions used in the oligotyping analysis, and final number of oligotypes obtained are included.

### Statistical Analyses

Analysis of variance (ANOVA) was used to assess statistically significant differences in environmental and abundance data. The “*aov*” function of the “*vegan*” package of R with a significance level of *p* < 0.05 was used. Distance-based redundancy analysis (db-RDA) was used to evaluate the prokaryotic community variation based on unweighted UniFrac distances. Only non-colinear variables were selected as explanatory variables based on the variance inflation factor (VIF, Zuur et al., 2009) analysis, i.e., temperature, salinity, nitrate, nitrite, phosphate, Chl-*a*, phaeopigments, station, season, depth, and year. PAR was not included in the analysis due to limited data availability (18 depth profiles out of a total of 31 were available; missing PAR values were due to night measurements or sensor malfunction). The explained variation and the significance of the explanatory variables were assessed using “*dbrda*” and “*anova.cca*” functions of the “*vegan*” package of R. Variation partitioning was applied to the db-RDA model to discern the variability explained by environmental, spatial, or temporal variables using “*varpart*” function of the “*vegan*” package of R. Environmental variables included temperature, salinity, and inorganic nutrients concentrations; spatial variables included depth and station; and temporal variables included season and year. Prokaryotic community was further analyzed using db-RDA analysis for surface and DCM communities separately. Due to collinearity, silicate and Chl-*a* were excluded from the surface model and nitrate was excluded from the DCM model. RDA was used to relate oligotypes composition with environmental parameters. Same explanatory variables selected for the db-RDA prokaryotic community model were used. Pearson correlation was used to reveal connectivity of oligotypes for surface and DCM communities separately based on their relative abundance. Significantly, correlated oligotypes (*p* < 0.05) were visualized in a network using the Cytoscape open-source platform (Shannon et al., 2003). Subsequently, the resulting networks were referred to as association networks, as they include both positive (co-occurrence, co-presence) and negative correlations (non-coexistence or exclusion).

## Results

### Physicochemical and Biological Temporal and Spatial Variability

Temperature ranged between 13 and 15°C throughout the water column in winter and increased to 16–17°C at surface in spring. During the summer stratification, surface temperature reached 23–26°C and remained at 13–15°C below the thermocline (>50 m). The thermocline was disrupted in autumn, with temperature ranging between 18–22 and 13–22°C at surface and below 50 m, respectively (Supplementary Figure S2A). Available PAR data did not show significant differences between seasons at surface (0–10 m), ranging between 4.2 and 79.5%. However, PAR reached 5.4 ± 1.6% (mean ± SE) at the DCM depth in winter and 1.9 ± 0.5% in the other seasons (Supplementary Figure S2E).

No significant differences in inorganic nutrient concentrations were found between winter surface and DCM waters, ranging 0–3.07, 0–0.20, 0.01–0.14, and 0.59–2.26 μM for nitrate, nitrite, phosphate, and silicate, respectively. Surface nutrient concentrations were significantly lower than DCM concentrations in spring, summer, and autumn, varying 0–0.05, 0–0.13, 0–0.04, and 0.37–1.15 μM for nitrate, nitrite, phosphate and silicate, respectively, at surface, and between 0.01–3.13, 0.01–0.27, 0–0.10, and 0.53–2.06 μM for nitrate, nitrite, phosphate, and silicate, respectively, at DCM (Supplementary Figure S3).

Water column maximum Chl-*a* fluorescence values were observed at increasing depths from winter to summer (Supplementary Figure S2D). Chl-*a* concentration ranged between 0.34 and 1.07 mg m^−3^ in winter and peaked at 0–30 m. In spring, Chl-*a* ranged between 0.23 and 1.55 mg m^−3^ with maximum at 37–40 m. Subsequently, Chl-*a* varied between 0.42 and 1.34 mg m^−3^ and its maximum deepened to 58–100 m in summer, rising up to 47–58 m depth in autumn with concentrations ranging between 0.20 and 1.07 mg m^−3^ (Supplementary Figure S2D).

### Seasonal Patterns of Microbial Communities

**Figure 1:**
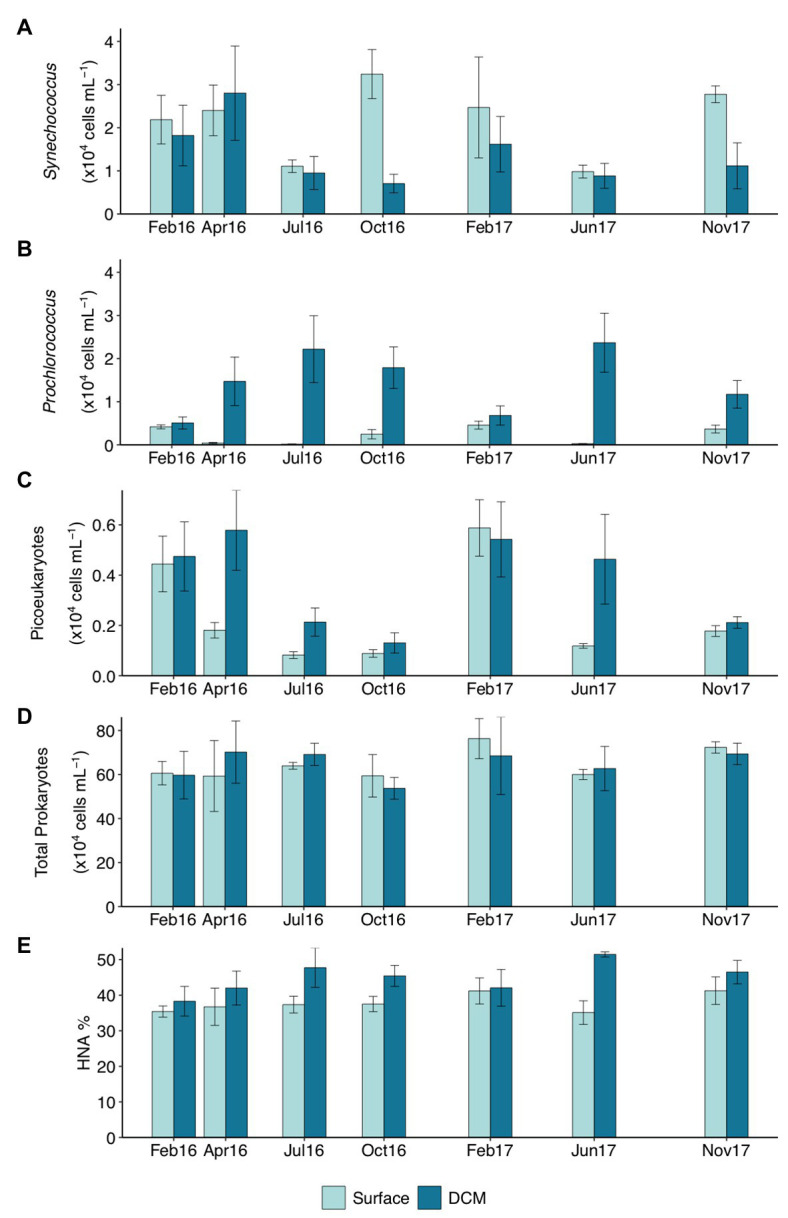
Abundance (×10^4^ cells ml^−1^) of **(A)** *Synechococcus*, **(B)** *Prochlorococcus*, **(C)** picoeukaryotes, **(D)** total prokaryotes, and **(E)** percentage of high nucleic acid (HNA) cells throughout the studied period in surface and deep chlorophyll maximum (DCM) waters. Notice different scales for **C**–**E**. Bar height indicates the corresponding average value of all the stations at the specific depth and campaign, vertical lines indicate the standard error. Feb16 and Feb17 correspond to winter season, Apr16 to spring, Jul16 and Jun17 to summer, and Oct16 and Nov17 to autumn. DCM, deep chlorophyll maximum.

Seasonality of picophytoplankton groups (Figure 1) was statistically significant when depth layer was considered (Season*Depth, *p* < 0.001, ANOVA). The geographic location (station) was not significantly related to abundance variations (*p* > 0.05, ANOVA). *Synechococcus* was more abundant at the upper 25 m in winter (Feb16 and Feb17) and autumn (Oct16 and Nov17) (2.67 ± 0.21 × 10^4^ cells ml^−1^, mean ± SE) compared to deeper layers (Figure 1A; Supplementary Figure S4). *Synechococcus* surface abundance (0–25 m) decreased to 0.86 ± 0.08 × 10^4^ cells ml^−1^ in summer (Jul16 and Jun17), concurrently increasing to 1.96 ± 0.36 × 10^4^ cells ml^−1^ at 50–75 m (Supplementary Figure S4). *Prochlorococcus* were always more abundant below 50 m than at upper layers, and increased significantly at DCM-75 m in summer (2.30 ± 0.43 × 10^4^ cells ml^−1^; Figure 1B; Supplementary Figure S4). Picoeukaryotes abundance peaked at surface and DCM during winter (4.99 ± 0.60 × 10^3^ cells ml^−1^) and at DCM in spring (Apr16, 5.78 ± 1.59 × 10^3^ cells ml^−1^), exhibiting lower abundances at other depths (on average 1.38 ± 0.19 × 10^3^ cells ml^−1^) and seasons (on average 1.84 ± 0.30 × 10^3^ cells ml^−1^) (Figure 1C; Supplementary Figure S4).

Total prokaryotic abundance decreased with depth, ranging between 6.40 ± 0.27 × 10^5^ cells ml^−1^ at surface and 2.21 ± 0.16 × 10^5^ cells ml^−1^ at 200 m. However, prokaryotes abundance increased in summer up to 9.84 × 10^5^ cells ml^−1^ at 50 and 75 m (Supplementary Figure S4). Relative abundance of HNA cells increased with depth from 37.61 ± 1.16% HNA at surface to 49.67 ± 1.52% HNA at 200 m (Supplementary Figure S4 and Supplementary Table S1). Surface HNA relative abundance was significantly different from DCM in summer (Figure 1E).

### Prokaryotic Community Composition

16S rRNA gene sequence analysis revealed a total of 6,727 ASVs. Fourteen percent of ASVs were shared between surface and DCM communities during the winter mixing period, decreasing to 10% in spring and to 4.3% in summer (Supplementary Figure S5). Subsequently, shared ASVs between surface and DCM increased to 11% in autumn (Supplementary Figure S5). Higher Shannon diversity index and evenness were observed in DCM than in surface communities (*p* < 0.01, ANOVA; Supplementary Figure S6). However, only surface communities showed significant seasonal diversity dynamics. Shannon index of surface prokaryotic communities peaked in winter and was minimum in summer (*p* < 0.05, ANOVA; Supplementary Figure S6).

**Figure 2:**
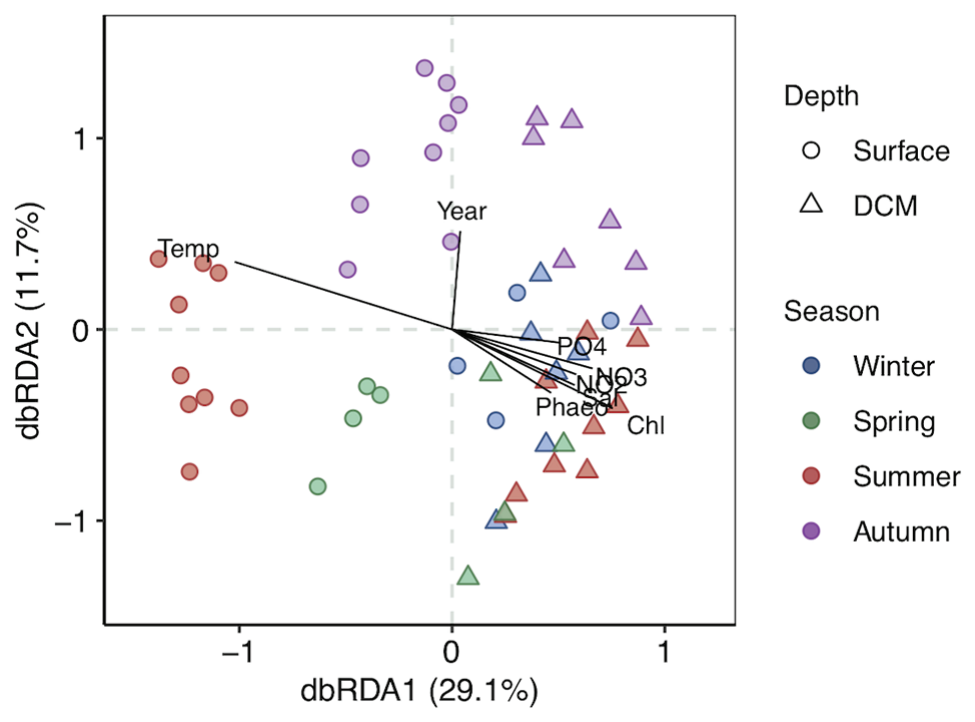
Distance-based redundancy analysis (db-RDA) based on unweighted UniFrac distances with constraint variables. The percentage of variance explained is shown for each axis. Vectors represent the constrained continuous variables used in the RDA model. Strength of relative correlations with RDA axes is indicated by the length and direction of vectors. Shapes indicate depth layer and colors indicate seasons.

Clustering of the communities according to depth (*p* < 0.05) and season (*p* < 0.01) was noticeable based on unweighted UniFrac distances (Figure 2). The first two RDA axes were significant (*p* = 0.001), explaining 40.8% of the constrained variation (Figure 2; Supplementary Table S2). Surface and DCM communities clustered together in winter and differed in other seasons, more pronouncedly in summer (Figure 2). Temperature and inorganic nutrients concentrations were significantly correlated to the first db-RDA axis (*p* < 0.01), whereas the sampling year (*p* < 0.05) correlated with the second db-RDA axis (Figure 2; Supplementary Table S2). Variation partitioning supported a main role of environmental variables on community composition, accounting for 67% of the explained variability, 33 and 17% of which was shared with spatial and temporal variability, respectively (Supplementary Figure S7). Community composition variability explained uniquely by environmental, temporal (season and year), and spatial (station and depth) variables separately accounted for 17, 17, and 4%, respectively (Supplementary Figure S7). The db-RDA analyses of surface and DCM communities separately indicated that temperature and season influenced significantly prokaryotic community structure throughout the photic zone (*p* < 0.01; Supplementary Figure S8 and Supplementary Table S3). Nitrite significantly affected surface communities (*p* < 0.05), while silicate (*p* < 0.05) and Chl-*a* (*p* < 0.01) were significant for DCM communities (Supplementary Figure S8 and Supplementary Table S3).

**Figure 3:**
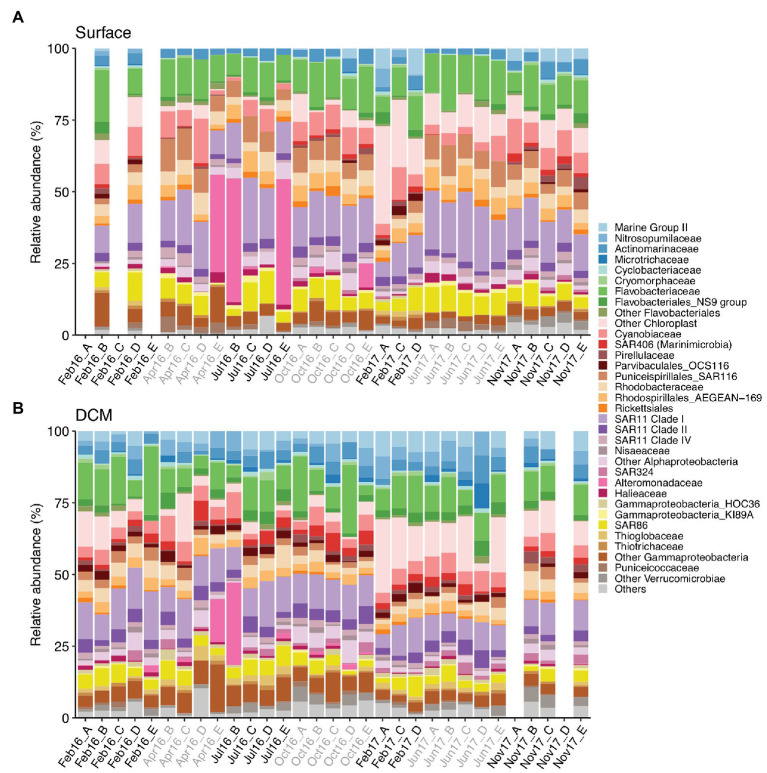
Prokaryotic community composition at family level at **(A)** surface and **(B)** DCM from February 2016 to November 2017. Samples are labeled according to cruise and station (Cruise_Station). Phylotypes contributing ≤0.6% are combined in “Others” group. Legend indicates order_family levels. Cruise correspondence to seasons as in Figure 1. DCM, deep chlorophyll maximum.

The photic zone prokaryotic communities were dominated by SAR11 Clade I, Flavobacteriaceae, and Cyanobiaceae, contributing 14.8% (±0.8), 12% (±0.6), and 6.7% (±0.6) at surface and 10.7% (±0.5), 10.4% (±0.9), and 4.8% (±0.3) at DCM [mean (±SE)], respectively (Figure 3). Chloroplast families contributed on average 7.4% (±1.4) at surface and 7.8% (±1.2) at DCM communities, increasing in winter to 17.7% (±4.8) and 11.1% (±2.8) at surface and DCM, respectively. SAR86 families also contributed considerably to photic zone communities on average 6.4% (±0.5) at surface and 4.1% (±0.3) at DCM. SAR116 (Puniceispirillales) were more abundant at surface (6.3 ± 0.5%) than at DCM (2.1 ± 0.2%) waters, peaking during spring and summer. Nitrosopumilales and Marine Group II families contributed more to DCM communities, 3.9% (±0.4) and 4.2% (±0.4), respectively, than to surface communities, contributing 0.5% (±0.2) and 1.5% (±0.4) on average, respectively (Figure 3).

Alteromonadaceae comprised on average 0.4% (±0.2) of the photic zone community. However, Alteromonadaceae increased drastically its contribution to definite spring-summer communities from surface and DCM, accounting for 15–43.7% of the community (Figure 3). Other family phylotypes considerably contributing to photic zone communities were Rhodobacteraceae (4.1 ± 0.2% at surface and 3.7 ± 0.3% at DCM), AEGEAN-169 group from Rhodospirillales (4.4 ± 0.3% at surface and 2.9 ± 0.2% at DCM), Actinomarinaceae (3.1 ± 0.2% at surface and 3.8 ± 0.3% at DCM), SAR11 Clade II (2.7 ± 0.1% at surface and 3.8 ± 0.2% at DCM), and Clade IV (2 ± 0.1% at surface and 1.3 ± 0.1% at DCM). OCS116 from Parvibaculales exhibited pronounced seasonal variation at surface (2.1 ± 0.3% in winter and 0.2 ± 0.1% in other seasons), whereas steady abundances at DCM were obtained throughout the study (1.9 ± 0.1%) (Figure 3).

### Oligotypes Composition

**Figure 4:**
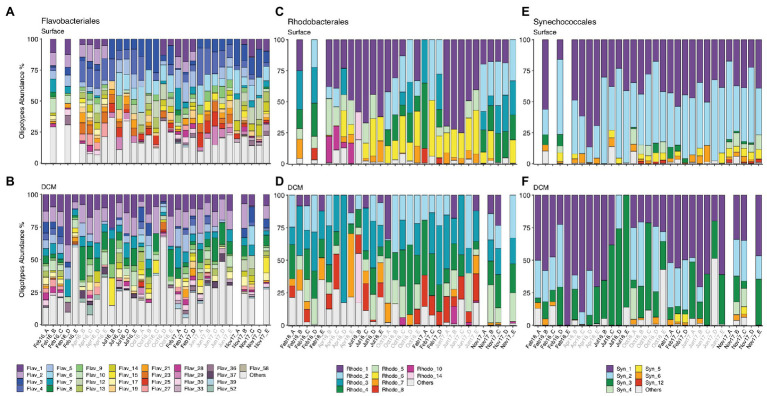
**(A,B)** Flavobacteriales, **(C,D)** Rhodobacterales, and **(E,F)** Synechococcales oligotype composition from surface and DCM from February 2016 to November 2017. Samples are labeled according to cruise and station (Cruise_Station). Cruise correspondence to seasons as in Figure 1. Legends indicate phylotype_oligotype number for each phylotype. Oligotypes that show no temporal pattern are combined in “Others” group. Missing bars indicate missing samples. DCM, deep chlorophyll maximum.

Oligotyping analysis revealed between 22 and 103 distinct oligotypes for the selected phylotypes examined (Table 1). Oligotypes composition of all phylotypes was remarkably different between surface and DCM (Figure 4; Supplementary Figure S9).

Flavobacteriales, Rhodobacterales, SAR11, Synechococcales, SAR86, and Actinomarinales exhibited seasonal patterns at surface communities, evidenced by appearance or disappearance of specific oligotypes as well as changes in relative abundance of the most abundant oligotypes in different seasons (Figure 4; Supplementary Figure S9). Flavobacteriales oligotypes 1 and 2 disappeared of surface samples during summer and autumn (Jul16, Oct16, Jun17, and Nov17), when stratification prevailed, while the relative abundance of other oligotypes increased (Figure 4A). Similarly, some surface Rhodobacterales oligotypes (e.g., oligotypes 2 and 4) retreated during summer stratification (Figure 4C). Synechococcales oligotypes composition varied seasonally at surface and DCM, linked to changes in low (e.g., oligos 3, 4, 5, 6, and 12) and high abundance oligotypes (e.g., oligos 1, 2, and 3), respectively (Figures 4E,F). SAR86 oligotypes 4, 5, and 8 increased in spring and summer (Apr16, Jul16, and Jun16), while oligotype 6 developed in autumn (Oct16 and Nov17) (Supplementary Figure S9E). Seasonal dynamics of Actino-marinales oligotypes composition was less pronounced than the previously mentioned groups, however, specific oligotypes developed in autumn (oligotypes 12–15; Supplementary Figure S9G). No clear temporal patterns were observed for Marine Group II, Chloroplast, or Alteromonadales oligotypes (Supplementary Figure S9).

The variability of oligotypes composition related to environmental parameters was tested with RDA (Supplementary Figure S10 and Supplementary Table S4). The first RDA axis was significant (*p* < 0.01) for the models of all groups, and the second axis was also significant (*p* < 0.01) for all the group models except Alteromonadales and Marine Group II (Supplementary Table S4). Depth (surface vs. DCM) and season significantly explained Flavobacteriales, Rhodobacterales, Synechococcales, SAR11, SAR86, Actinomarinales, and Marine Group II oligotypes composition. Surface and DCM oligotypes composition grouped together in winter and clustered separately in summer except for Alteromonadales oligotypes composition (Supplementary Figure S10). Besides, nitrate concentration significantly explained Flavobacteriales, SAR11, and SAR86 oligotypes composition and phosphate concentration contributed to explain the variability of Rhodobacterales, Synechococcales, SAR11, and Marine Group II oligotypes composition. Nitrite, Chl-*a*, and phaeopigments also significantly explained the variability of SAR11 oligotypes composition. The location of the samples (station) significantly accounted for Synechococcales, SAR86 and Alteromonadales oligotypes composition changes but not for the oligotypes composition of the other groups (Supplementary Figure S10 and Supplementary Table S4).

### Network Association Analysis of Oligotypes

Relative abundances of phylotypes Rhodobacterales and SAR86 and of Marine Group II and Chloroplast at order level significantly correlated both at surface and DCM (*p* < 0.05, ANOVA; Supplementary Figure S11). Relative abundances of the other orders analyzed were only significantly correlated at surface, e.g., Synechococcales and Rhodobacterales, SAR11 and SAR86 or Rhodobacterales and SAR11 (*p* < 0.05, ANOVA; Supplementary Figure S11A).

**Figure 5:**
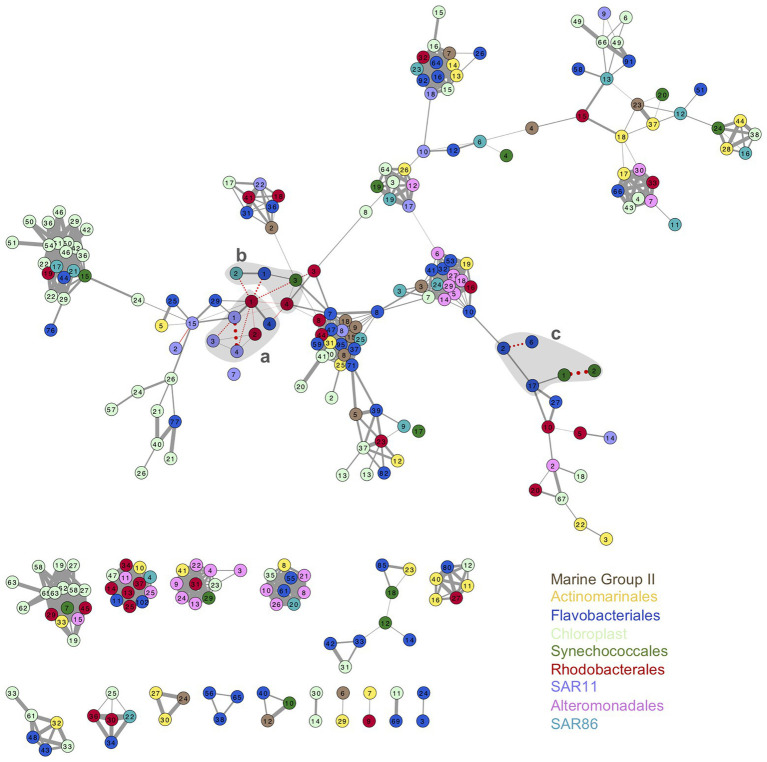
Association network of surface oligotypes. Each node represents one oligotype, node colors indicate the nine phylotypes studied (see legend). Width of edges connecting nodes is drawn proportionally to the Pearson correlation value between the two oligotypes. Solid gray edges indicate positive correlations and pointed red edges indicate negative correlations. Only significant correlations are shown (*p* < 0.05). Shaded gray areas labeled a, b, and c enclose the association of oligotypes represented in Figure 6 and Supplementary Figure S13.

Association networks of oligotypes from all the phylotypes analyzed showed different connectivity patterns for surface and DCM (Figure 5; Supplementary Figure S12). Association network from surface oligotypes exhibited lower interconnectivity (Figure 5) than DCM oligotypes (Supplementary Figure S12). Separated assemblies in surface communities were comprised mostly by low abundance oligotypes concurrently occurring in one or few discrete samples, which resulted in high correlation coefficients (Figure 5).

**Figure 6:**
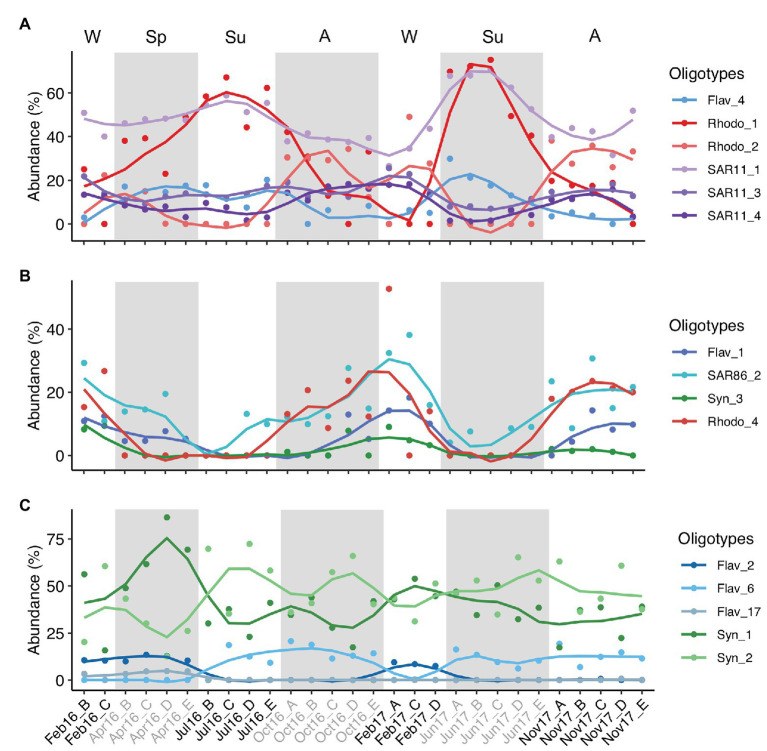
Temporal variation of three groups of connected abundant oligotypes. The three groups of oligotypes **(A–C)** correspond to those enclosed in shaded gray areas in surface network of Figure 5. Y-axis indicates the relative abundance of the oligotypes analyzed in this study. Lines are loess smoothed (smoothing function to facilitate trends visualization) and dots indicate the data values. Samples are labeled according to cruise and station (Cruise_Station). Alternate light/shaded areas indicate changing seasons: W (winter), Sp (spring), Su (summer), and A (autumn). Oligotypes are labeled according to phylotype_oligotype number. Flav, Flavobacteriales; Rhodo, Rhodobacterales; Syn, Synechococcales.

As example of seasonal dynamics of connected oligotypes within and between phylotypes, we selected a set of abundant oligotypes from surface communities surrounded by gray areas in Figure 5. The two most abundant oligotypes from Rhodobacterales and Synechococcales (oligotypes 1 vs. 2) exhibited opposed seasonal patterns at surface waters (Figures 6A,C). Rhodobacterales oligotype 1 dominated in spring and summer while oligotype 2 increased in winter and autumn (Figure 6A). Synechococcales oligotype 1 dominated in winter and spring and oligotype 2 dominated in summer and autumn (Figure 6C). However, although also negatively correlated, oligotypes 1 and 2 from Rhodobacterales and Synechococcales did not show the same temporal patterns at DCM (Supplementary Figures S13A,C). Similarly, SAR11 oligotype 1 exhibited an opposed seasonal pattern to that of oligotypes 3 and 4 (Figure 6A), whereas Flavobacteriales oligotype 6 displayed opposed seasonal dynamics to oligotypes 2 and 17 at surface waters (Figure 6C). However, neither SAR11 nor Flavobacteriales oligotypes showed clear seasonal trends at the DCM (Supplementary Figure S13).

Inter-phylotype connection of abundant oligotypes was noticeable at surface waters, e.g., Flavobacteriales oligotype 1, SAR86 oligotype 2, Synechococcales oligotype 3, and Rhodobacterales oligotype 4 relative abundances decreased during summer and increased in winter and autumn (Figure 6B). However, these oligotypes showed different temporal patterns at DCM communities (Supplementary Figure S13B). Marine Group II, Chloroplast, Alteromonadales, and Actinomarinales oligotypes co-occurred with other low-abundant oligotypes in the association networks (Figure 5; Supplementary Figure S13).

## Discussion

### Seasonal and Spatial Dynamics of Prokaryotic Communities in the Western Mediterranean Sea

The upper-ocean prokaryotic communities were strongly shaped by winter water column mixing and summer stratification in agreement with previous findings in the open ocean and coastal waters (Treusch et al., 2009; Gilbert et al., 2012; Fuhrman et al., 2015; García et al., 2015; Salter et al., 2015). Noticeably, surface prokaryotic communities are subjected to more pronounced seasonal variability than DCM communities. The different seasonal trends between surface and DCM are driven by the different variability in seawater temperature between the two depths, as well as the nutrient depletion in summer surface waters associated to thermal stratification (Pinhassi et al., 2006; D’Ortenzio and Prieur, 2012). Accordingly, surface communities markedly differed from DCM communities during summer stratification, separated by the thermocline structure, whereas during the winter mixing, surface and DCM communities were more similar and shared a higher proportion of ASVs (Figure 2; Supplementary Figure S5; Morris et al., 2005; García et al., 2015; Salter et al., 2015). Hence, irradiance intensity, primarily governing surface ocean temperature, and nutrient availability are key factors shaping the seasonal and depth-related trends of microbial communities in marine systems, even in ocean regions with mild climatic variability, such as the North Pacific Subtropical Gyre (Bryant et al., 2016; Mende et al., 2017).

The seasonal variability of the epipelagic Mediterranean Sea microbial communities was prominent for the autotrophs *Synechococcus* and *Prochlorococcus*. Depth distribution patterns of these two phototrophs are strongly influenced by irradiance levels and light stress responsiveness (Mella-Flores et al., 2012). The relative abundance of *Synechococcus* and *Prochlorococcus* decreased at the highly irradiated surface waters and the two taxa sheltered below the thermocline in summer. This finding supports the negative effect of light and radiation overexposure on the growth of these phototrophs, *Prochlorococcus* being more sensitive to excess radiation (Mella-Flores et al., 2012). The temporal and depth variability of these two picophytoplankters contrasts with other findings in the oligotrophic Atlantic and Pacific oceans. *Prochlorococcus* dominates picophytoplankton abundance in the photic zone at the Bermuda Atlantic Time-Series Study (BATS) site, except during the spring bloom mixing period, when similar abundances of *Prochlorococcus* and *Synechococcus* occur (DuRand et al., 2001). At the North Pacific Subtropical Gyre (station ALOHA), *Prochlorococcus* dominates year-round without remarkable seasonal changes (Bryant et al., 2016). Considering that *Synechococcus* and *Prochlorococcus* dominate the photosynthetic community in the oligotrophic Mediterranean Sea (Mena et al., 2019), the results suggest an important role of these groups in the strong primary production seasonality of the open ocean Mediterranean Sea (Marty and Chiavérini, 2002), with reportedly higher new primary production at the DCM during summer stratification and in the upper layers during mixing conditions (Estrada, 1996; Pedrós-Alió et al., 1999). Based on ocean color and a phytoplankton class-specific bio-optical model, Uitz et al. (2012) estimated an annual contribution of picophytoplankton of ~31% in the western basin of the Mediterranean Sea, smaller than nanophytoplankton (~48%) and larger than microphytoplankton (~21%). However, contribution of picophytoplankton to primary production changes according to local environmental conditions (Magazzu and Decembrini, 1995) and seasonally (Charles et al., 2005). Further research on the temporal and spatial activity of these two autotrophs and the comparison to the activity rates of eukaryotic phototrophs are needed to complete our view of the carbon cycle in the upper Mediterranean Sea.

Similarly to the cyanobacteria groups, uncultured Alphaproteo-bacteria OCS116 and Marine Group II group revealed similar contributions at surface and DCM during the mixed period, which persisted at the DCM throughout the study, in agreement with previous findings on seasonal trends of these phylotypes (Morris et al., 2005; Treusch et al., 2009; Hugoni et al., 2013; Haro-Moreno et al., 2018). OCS116 had been described to concurrently bloom with phytoplankton in spring (Morris et al., 2005; Treusch et al., 2009); however, no positive correlation to Chl-*a* was found in this study.

Contrary to the previous groups, SAR116 clade members thrived in the stratified surface waters in agreement with their association with warmer temperatures and water stratification (Morris et al., 2005; Giovannoni and Vergin, 2012; Alonso-Sáez et al., 2015; Bryant et al., 2016). SAR116 clade members harbor a diverse metabolic potential, including dimethylsulfoniopropionate degradation, oxidation of one-carbon compounds, potential tolerance to high irradiance levels, and their own light-dependent proteorhodopsin (Oh et al., 2010), suggesting an important role in the carbon dynamics of the oligotrophic Mediterranean Sea during the stratified period. Other groups show no apparent seasonal variations, such as orders Flavobacteriales or Rhodobacterales (Figure 3; Supplementary Figure S11), abundant photic groups frequently associated with phytoplankton blooms (Buchan et al., 2014). Also, no significant temporal patterns for SAR11 and SAR86 clades were determined in our study (Figure 3; Supplementary Figure S11), in contrast to previous studies in the Sargasso Sea (Morris et al., 2005; Treusch et al., 2009) and NW Mediterranean Sea (Salter et al., 2015). However, these groups harbor a large intra-diversity and consist of numerous ecotypes adapted to different environmental conditions (Vergin et al., 2013a; Hoarfrost et al., 2019), as discussed further on.

### Seasonality of Oligotypes Within Particular Phylotypes

The function and dynamics of an ecosystem is contingent on the assembly of taxa playing ecologically distinct roles (ecotypes) and on their interactions with other taxa and with the environment (Koeppel et al., 2008). A recent approach to determine the presence and dynamics of ecotypes within closely related taxa is oligotyping (Eren et al., 2013). Indeed, Flavobacteriales, Rhodobacterales, SAR11, and SAR86 showed seasonal dynamics only at oligotype level in this study (Supplementary Figure S10) but not at order level (Figure 3; Supplementary Figure S11). Although the main differences were observed between winter and summer, i.e., under well-defined mixed and stratified conditions (Salter et al., 2015), there were particular oligotypes preferentially associated to spring (e.g., Rhodobacterales oligotype 10; Figure 4C) or autumn conditions (e.g., Flavobacteriales, Synechococcales, SAR86, and Actinomarinales oligotypes; Figures 4A,E; Supplementary Figure S9). SAR86 clade, significant contributor to the surface ocean communities (Dupont et al., 2012; this study), showed notable seasonal variability in oligotype composition and a diversity decrease during winter at surface (Supplementary Figure S9E).

The temporal patterns of oligotypes suggest niche specialization. Ecotypes differentiation can be originated by spatial and temporal environmental gradients. Temperature and light-level induce depth differentiation of *Prochlorococcus* ecotypes (Zwirglmaier et al., 2008). The absence of specific oligotypes at surface during the stratified period (e.g., oligotypes 3 and 4 of Synechococcales; Figure 4E) could be explained by their sensitivity to light-stress and/or to low nutrient concentrations. Indeed, phosphate concentrations significantly explained the variability of Synechococcales oligotypes composition, suggesting that phosphate availability selects for distinct surface oligotypes during the stratification period, in accordance with the general phosphate limitation of the Mediterranean Sea (Pasqueron de Fommervault et al., 2015). Dissolved organic matter (DOM) is the largest reservoir of reduced carbon in the ocean (Hansell et al., 2009), supporting heterotrophic microbes throughout the water column (Kujawinski, 2011). However, DOM is composed of a very diverse and complex mixture of compounds, with different chemical and physical properties (Nagata, 2008), providing a heterogeneity of potential niches for heterotrophs. Fine-tuning of the metabolic capacities of closely-related microbes to the use of these diverse set of substrates helps explain the seasonality and spatial distribution of ecotypes within specific taxa (Carlson et al., 2009; Hehemann et al., 2016). Temporally recurring biotic and abiotic conditions, e.g., phytoplankton blooms and their associated production, atmospheric deposition (Koçak et al., 2010), or quantity and quality of organic carbon inputs (Pasqual et al., 2015), induce seasonal variations in availability of specific sets of DOM compounds for surface heterotrophic prokaryotic communities, supporting the recurring seasonal patterns of closely related taxa determined in coastal marine systems (Ward et al., 2017; Chafee et al., 2018) and in open Mediterranean sea waters (e.g., oligotypes 1 and 2 of Flavobacteriales or oligotypes 2, 3, and 4 of Rhodobacterales in this study).

Nevertheless, not all examined taxa exhibited clear seasonality of oligotypes. Presumably, the low abundance of sequences assigned to Marine Group II at surface and to Chloroplast in the first year of study hindered the detection of oligotypes and their potential temporal patterns. During the second year of study, Chloroplast sequences were more abundant, and seasonal differences in oligotypes diversity and composition during winter, summer, and autumn were determined. However, further studies will be needed to support recurring seasonal patterns for this group. The copiotrophic lifestyle of Alteromonadales resulted in a conspicuous feast and famine response (Vergin et al., 2013b). Their low abundance (or undetectability) in most samples interfered with oligotyping analysis, hindering the description of oligotypes composition, and therefore preventing to determine temporal variations. However, the pronounced increase in the total abundance of Alteromonadales in particular samples coincided with a notable increase of oligotypes diversity, indicating microdiversity of the blooming Alteromonadales. Albeit challenging, further studies focusing on disentangle the influence of environmental variability, substrate specialization, and predation dynamics on the differentiation of ecotypes are essential to understand microdiversity patterns and their influence in the metabolic activity of microbial communities (Alonso-Sáez and Gasol, 2007; Teira et al., 2019).

### Temporal Co-occurrence of Particular Phylotypes

Microbes interact in various ways with positive, negative, or neutral effects for one or both organisms implicated (Faust and Raes, 2012). Consequently, inter-organism interactions contribute to determine the realized niche of a specific organism and shape ecosystem function with profound effects on the biogeochemical ocean cycles (Strom, 2008). Oligotypes of different taxa showed temporal co-occurrence or exclusion patterns, however, the inter-oligotypes associations differed noticeably between surface and DCM communities, suggesting an influence of environmental conditions on the microbe-interactions and dynamics between the two depth layers (Cui et al., 2019) and the fine-tuning of the realized niche of closely-related taxa.

Surface communities are exposed to more variable conditions than DCM communities. During summer, surface Mediterranean prokaryotic communities are exposed to unfavorable conditions, including nutrient depletion and light stress (Ruiz-González et al., 2012; Tanhua et al., 2013). Microbes with an oligotrophic lifestyle have an advantage in nutrient poor environments; however, streamlining of their genomes induces the lack of essential metabolic pathways to synthesize key metabolites (Giovannoni et al., 2014) or to remove toxic or reactive molecules (e.g., Morris et al., 2011). Consequently, streamlined microorganisms depend on co-occurring microbes (Morris et al., 2011; Peura et al., 2015), successively resulting in the expansion of their realized niches and habitat ranges (Ma et al., 2018). Accordingly, free-living bacteria, generally harboring oligotrophic lifestyles, exhibit higher connectivity to other organisms than typically copiotrophic particle-attached bacteria (Milici et al., 2016).

The positive correlation between the total abundances of Rhodobacterales and SAR86 observed in this study suggests that the simpler compounds released by Rhodobacterales during the algal-derived organic matter degradation can be subsequently used by SAR86 (Dupont et al., 2012). Correlation between Rhodobacterales and SAR11 at surface was found not only at the order level but it was also substantiated by the seasonal correlation of particular surface ocean Rhodobacterales and SAR11 oligotypes. Oligotype 1 of both lineages increased during summer while oligotype 2 of Rhodobacterales and oligotypes 3 and 4 of SAR11 dominated during autumn-winter (Figure 6A). Rhodobacterales members perform key metabolic pathways involved in phytoplankton-derived organic matter processing (Buchan et al., 2014), resulting in the production and release of derived compounds which can be used by SAR11 members (Reintjes et al., 2019). Rhodobacterales have been described as a remarkably metabolically versatile group based on the genomic analysis of different isolates (Newton et al., 2010), supporting contrasting specific linkages between Rhodobacterales and SAR11 oligotypes in different seasons. Network associations have to be interpreted with caution (Faust and Raes, 2012), and the good agreement between the temporal dynamics of these specific phylotypes indicates either specialized interactions or similar ecological niche (e.g., utilization of similar substrates, similar effect of environmental factors, and/or interactions with other organisms such as predation/parasitism). Furthermore, our findings are based on a relatively low temporal sampling frequency (i.e., once per season) over approximately 2 years. Higher sampling resolution and extended time series studies should improve the robustness of the results and provide further support to the conclusions.

The seasonal dynamics of oligotypes also supports that the seed bank theory (Lennon and Jones, 2011) and the Baas Becking tenet (De Wit and Bouvier, 2006) applies to ecotypes within specific microbial taxa. Whereas Synechococcales oligotype 1 was dominant at DCM year-round with few exceptions and at surface from winter to spring, oligotype 2 dominated at surface in summer, indicating a better fitness of the latter oligotype under the summer environmental conditions (Figure 6; Supplementary Figure S13). Flavobacteriales oligotype 6 is a clear example of low abundance oligotype blooming when conditions are favorable in summer and autumn (Figure 6C).

Taken together, our results indicate a major effect of the seasonal stratification on prokaryotic community composition and diversity in the sunlit temperate open ocean. Noticeably, the temporal dynamics and association networks strongly responded to stratification conditions at a fine-scale phylogeny level, with implications for the future ocean function under the predicted increase of surface ocean temperatures and stratification associated to climate change. Oligotype dynamics within a specific taxon and connectivity patterns to other taxa oligotypes suggests temporal niche partitioning and fine-tuning of the realized niche of ecotypes, highlighting the importance of examining communities beyond common phylogenetically assigned taxa (Louca et al., 2016), and contributing to explain the plankton paradox. Further analysis on the metabolic functions of the different ecotypes using omic approaches will help elucidate how these oligotypes are specifically linked, and how their predicted dynamics in the framework of climate change might affect the global element cycles.

## Data Availability Statement

The datasets presented in this study can be found in online repositories. The names of the repository/repositories and accession number(s) can be found in the article/Supplementary Material.

## Author Contributions

CM, PR, RB, and ES conceived and planned the work. CM, RS, MM, and ES carried out the sampling and laboratory work. CM analyzed the data. CM and ES wrote the first draft of the manuscript. All authors contributed to the article and approved the submitted version.

### Conflict of Interest

The authors declare that the research was conducted in the absence of any commercial or financial relationships that could be construed as a potential conflict of interest.

## Footnotes

^1^http://qiime2.org
