# Supplementary material for: Seasonal Niche Partitioning of Surface Temperate Open Ocean Prokaryotic Communities
Source: Front Microbiol. 2020 Jul 28;11:1749. doi: 10.3389/fmicb.2020.01749 (PMC7399227; doi:10.3389/fmicb.2020.01749)
Supplement: Supplementary file 1 [file Data_Sheet_1.PDF]

Supplementary Material

Seasonal Niche Partitioning of Surface Temperate Open Ocean Prokaryotic Communities

Catalina Mena*, Patricia Reglero, Rosa Balbín, Melissa Martín, Rocío Santiago, Eva Sintes

*** Correspondence:** c.mena.oliver@gmail.com

1 Supplementary Figures

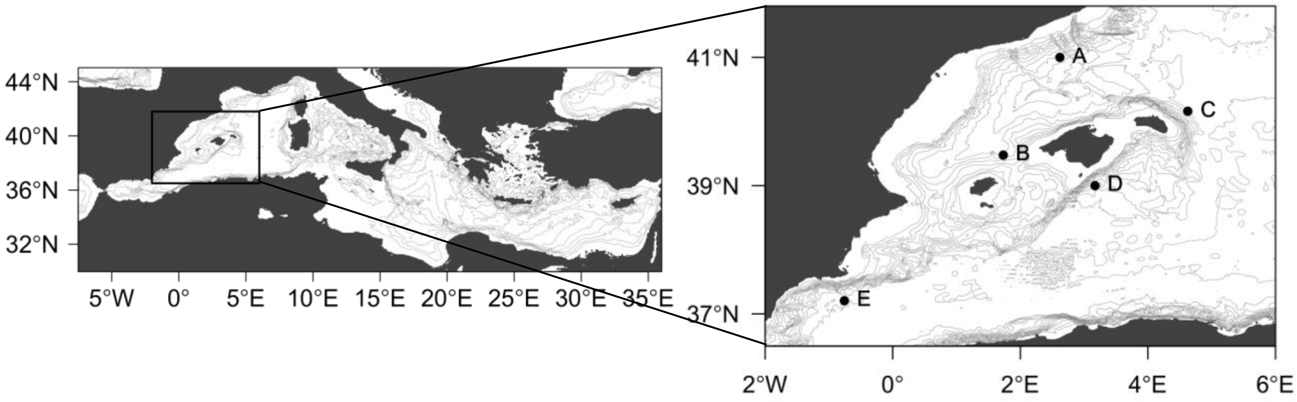

Supplementary Figure S1. Map of the Mediterranean Sea showing the stations sampled in the western basin. The stations were located at different regions: A at north Balearic sub-basin, B at the Mallorca channel, C at south Balearic sub-basin, D at north Algerian sub-basin and E at south Algerian sub-basin.

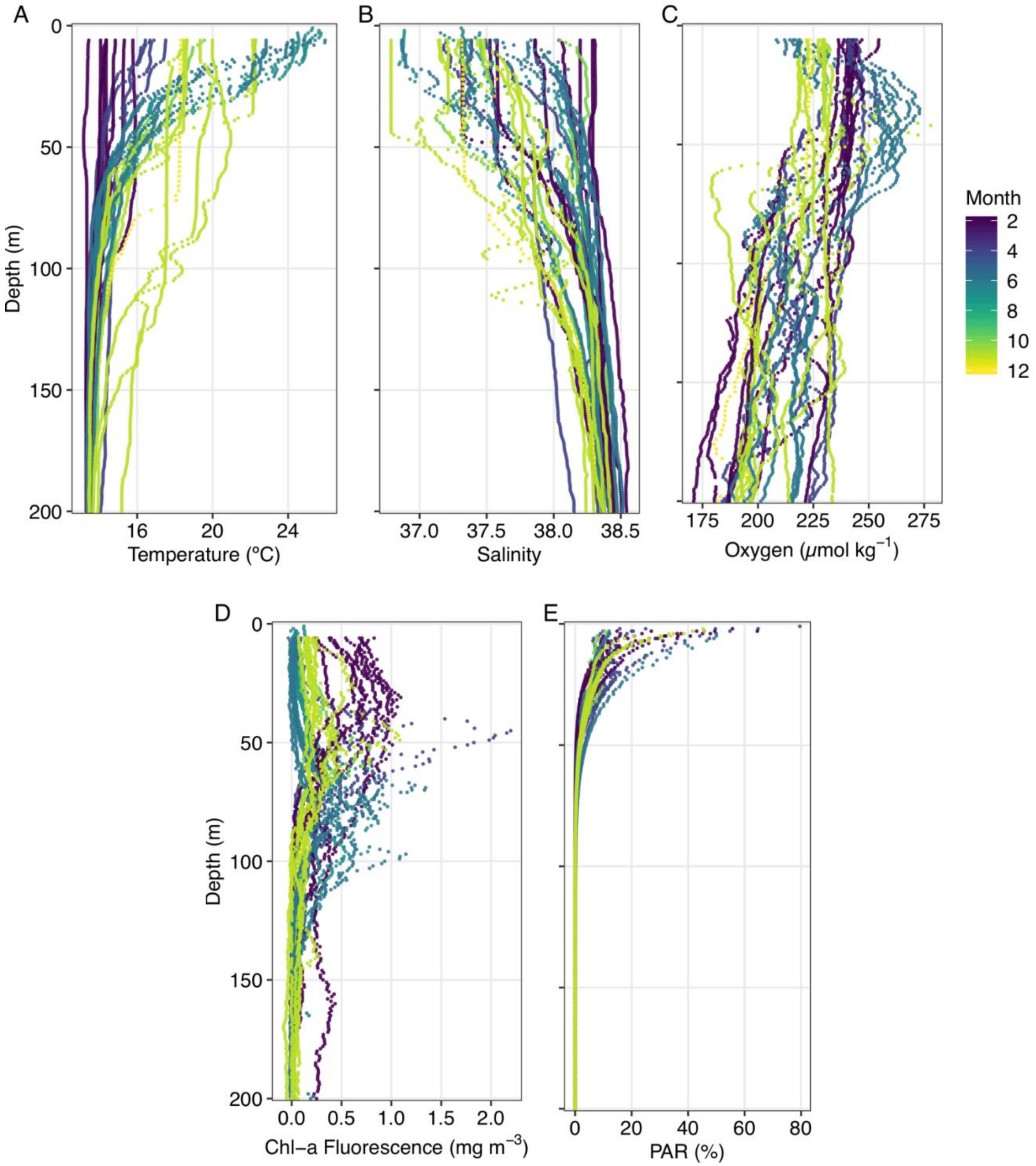

Supplementary Figure S2. Vertical profiles of (A) temperature, (B) salinity, (C) oxygen, (D) Chl-*a* fluorescence and (E) PAR throughout the upper 200 m of the western Mediterranean Sea. Color bar indicate month of year.

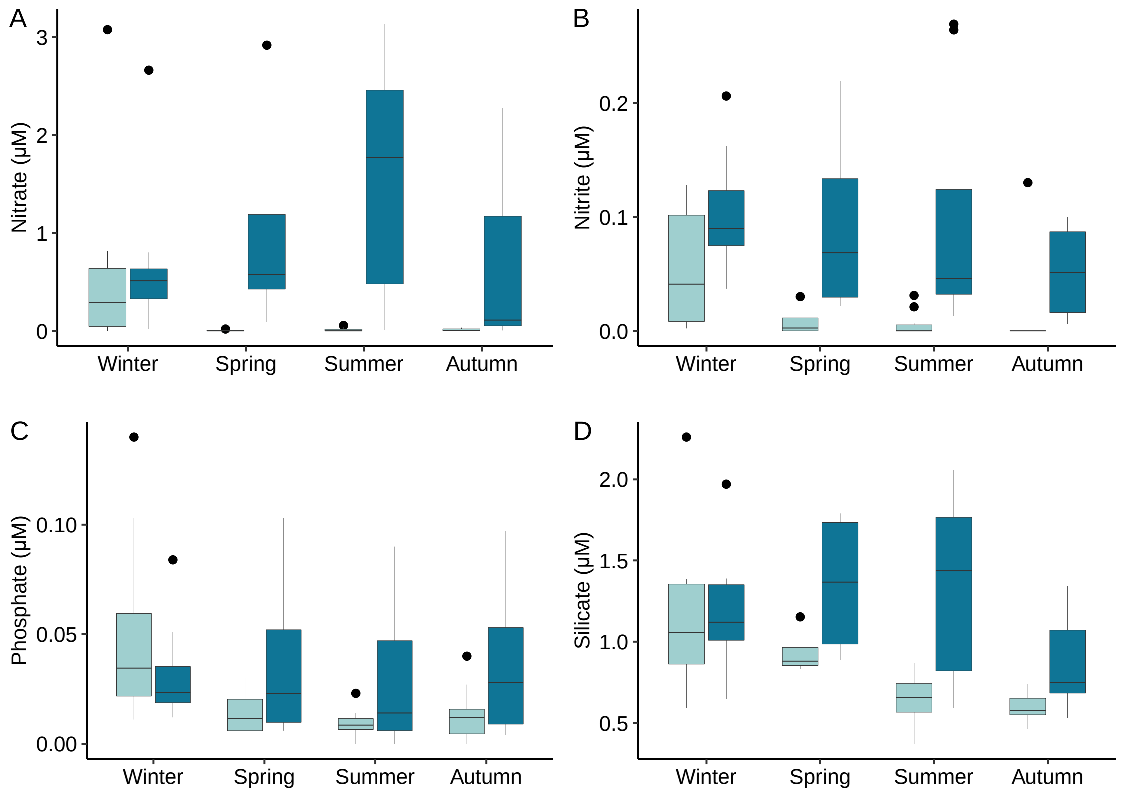

Supplementary Figure S3. Boxplots showing median inorganic nutrient concentrations in different seasons at surface (light blue) and DCM waters (deep chlorophyll maximum) (dark blue): (A) nitrate, (B) nitrite, (C) phosphate and (D) silicate. Median (line inside the boxes), first and third quartiles (upper and lower limits of boxes) and variability outside the first and third quartiles (extending vertical lines) are indicated. Black dots indicate outliers and gray diamonds indicate jittered values.

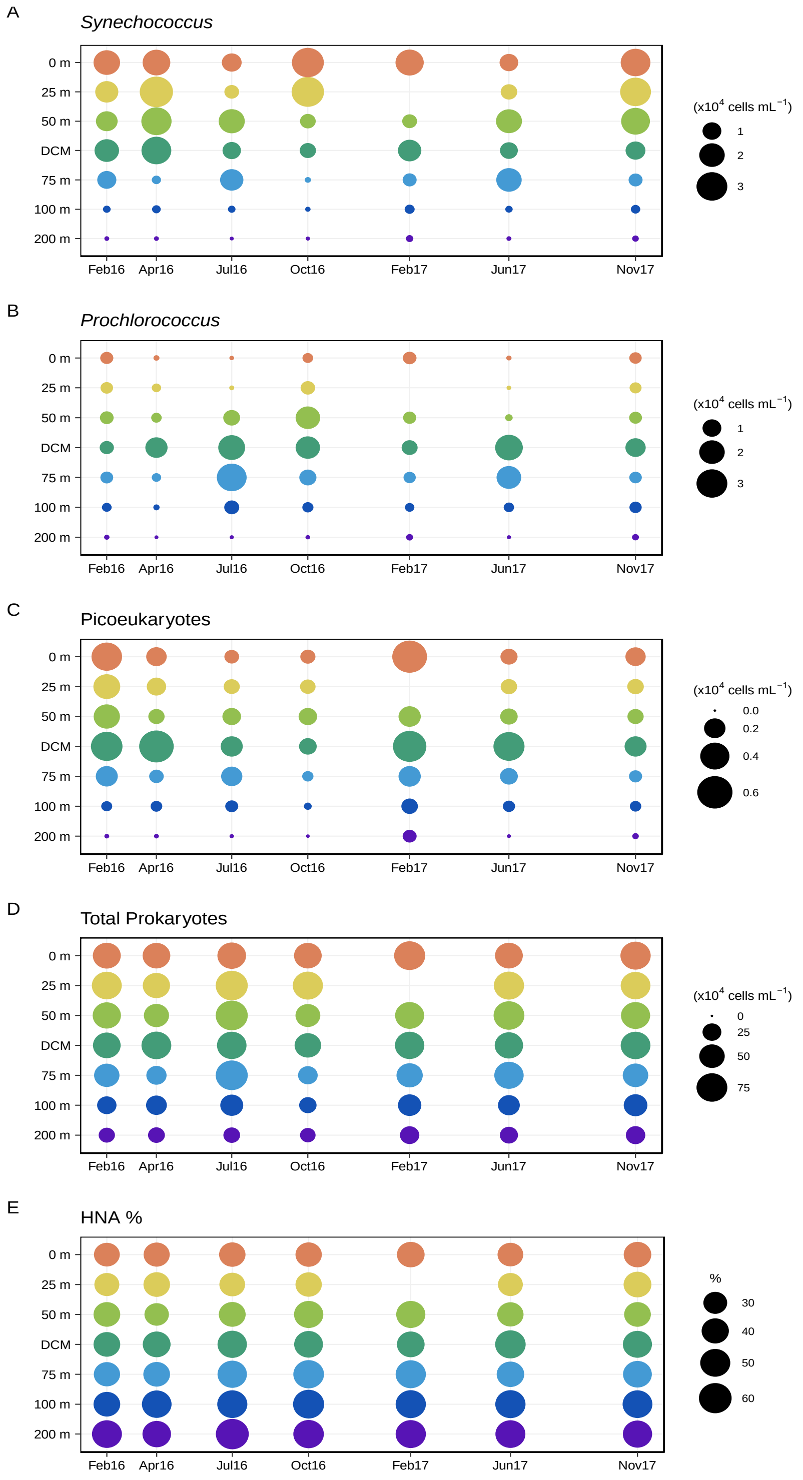

Supplementary Figure S4. Abundance (×10^4 cells mL^{-1}) of (A) *Synechococcus*, (B) *Prochlorococcus*, (C) picoeukaryotes and (D) total prokaryotes and (E) percentage of HNA cells throughout the studied period. Notice different scales for C, D and E. Color indicate the different depths. Average of all stations at the specific depth and campaign is shown. DCM: deep chlorophyll maximum.

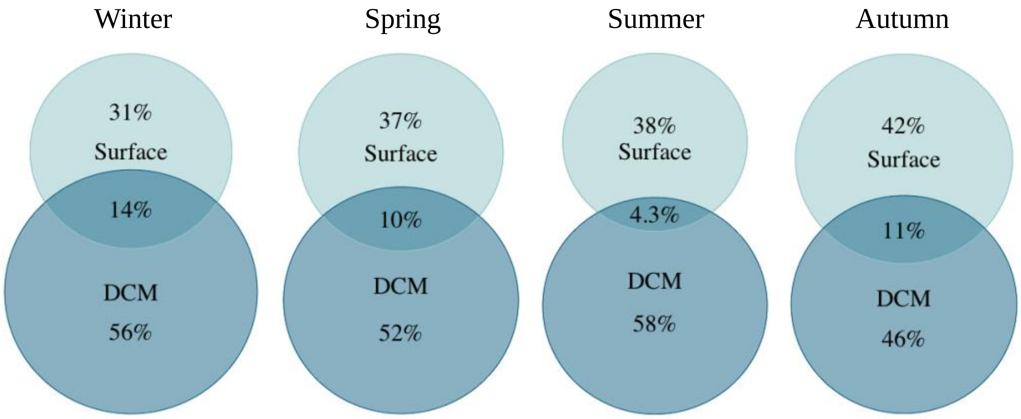

Supplementary Figure S5. Venn diagrams showing the percentage of unique and shared ASVs between surface and DCM (deep chlorophyll maximum) prokaryotic communities in different seasons in the Mediterranean Sea.

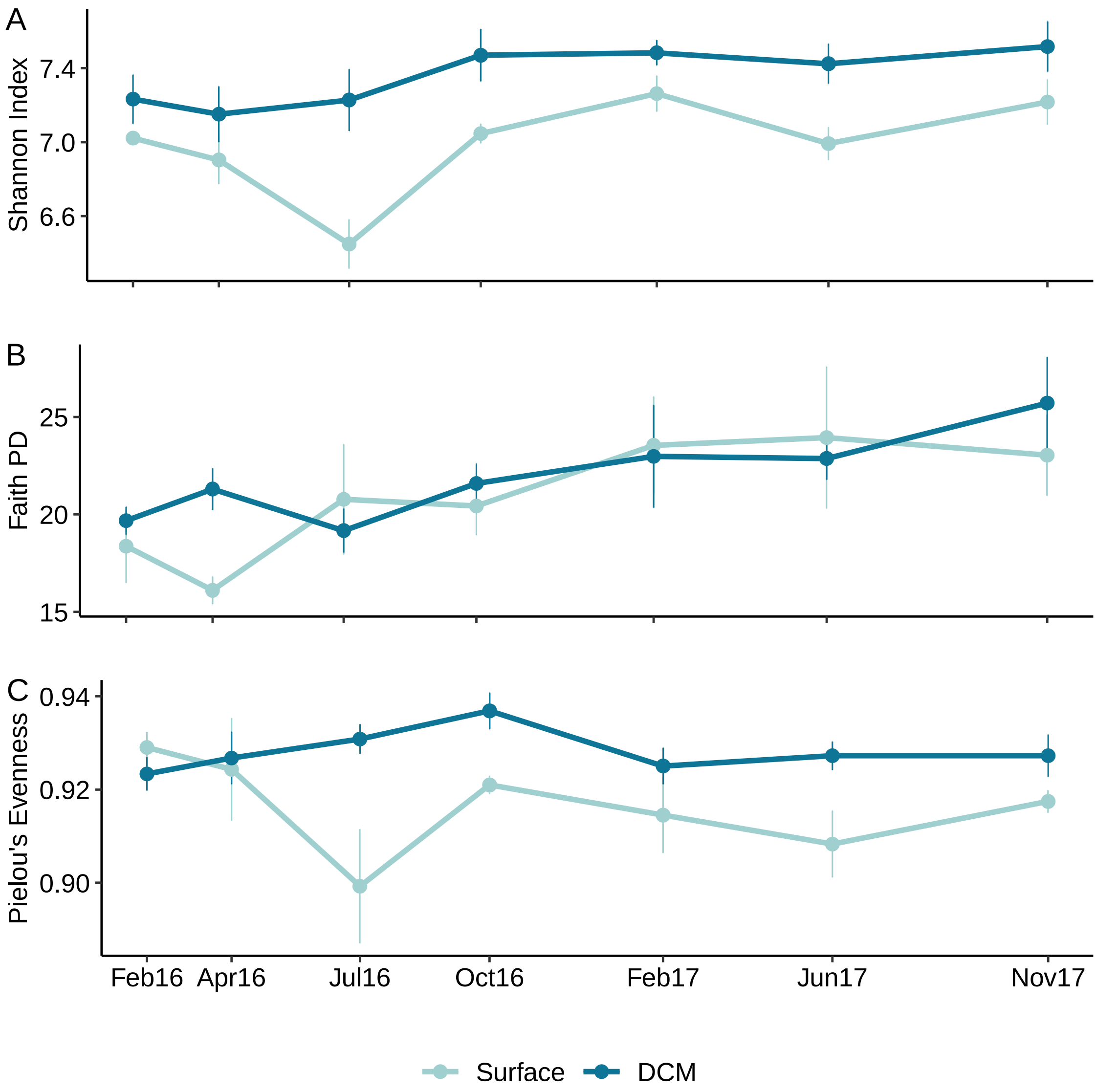

Supplementary Figure S6. Mean diversity and evenness indexes of surface and DCM prokaryotic communities throughout the sampling period in the Mediterranean Sea: (A) Shannon index, (B) phylogenetic diversity (Faith PD) and (C) Pielou's evenness. Cruise correspondence to seasons as in Fig. 1. Symbols indicate the average of all the stations at the specific depth and campaign, vertical lines indicate the standard error. DCM: deep chlorophyll maximum.

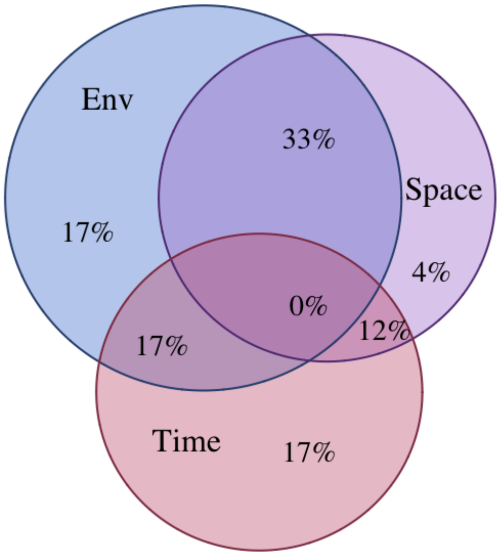

Supplementary Figure S7. Contribution of *Env* (environmental variables, including temperature, salinity and inorganic nutrients concentrations), *Space* (spatial variables, including depth and station) and *Time* (temporal factors, including season and year) to the constrained variation of the prokaryotic communities composition based on distance-based redundancy analysis (db-RDA) model.

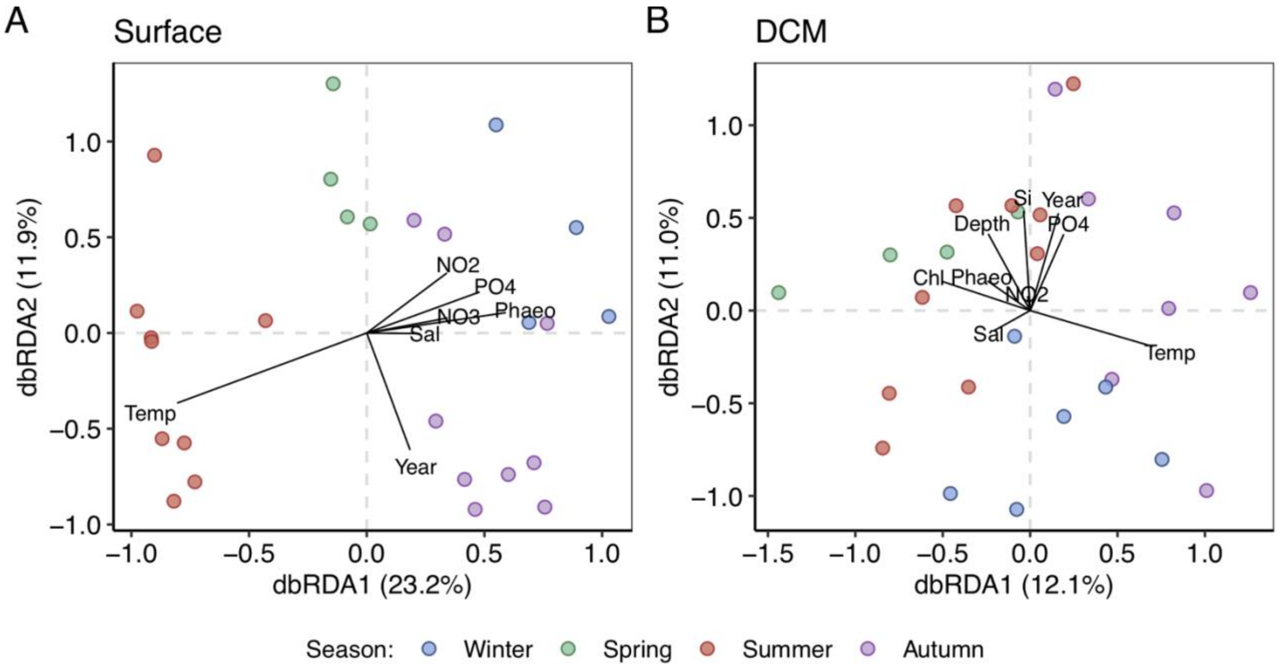

Supplementary Figure S8. Distance-based redundancy analysis (db-RDA) for (A) surface and (B) deep chlorophyll maximum (DCM) communities based on unweighted UniFrac distances with constraint variables. The percentage of variance explained is shown for each axis. Vectors represent the constrained continuous variables used in the RDA model. Strength of relative correlations with RDA axes is indicated by the length and direction of vectors. Colors indicate seasons. Temp: temperature; Sal: salinity; Chl: chlorophyll-*a*; Phaeo: phaeopigments.

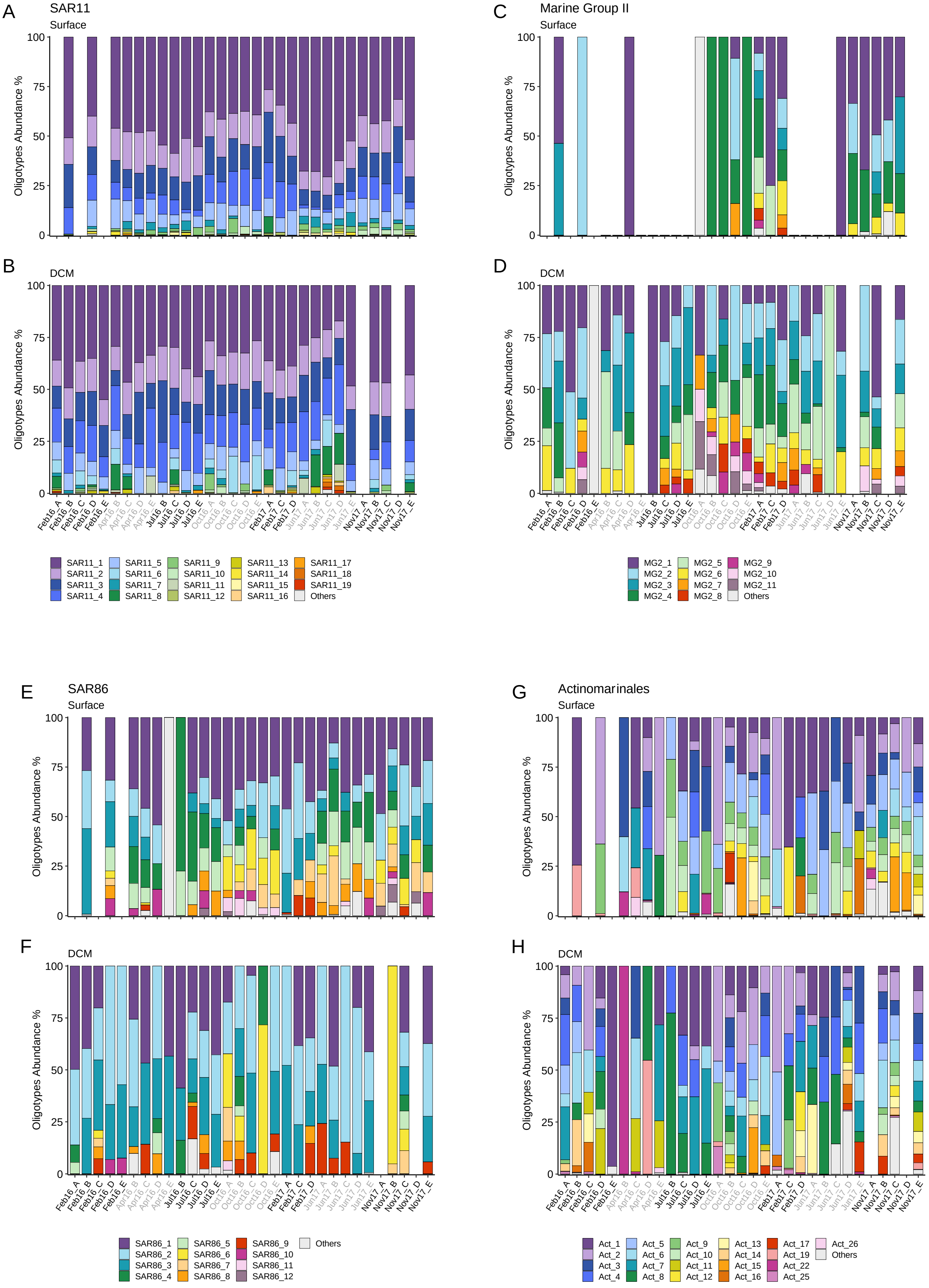

A
SAR11
Surface
B
DCM
Oligotypes Abundance %
Feb16_A Feb16_B Feb16_C Feb16_D Feb16_E Apr16_B Apr16_C Apr16_D Apr16_E Jul16_B Jul16_C Jul16_D Jul16_E Oct16_A Oct16_B Oct16_C Oct16_D Oct16_E Feb17_A Feb17_C Feb17_D Jun17_A Jun17_B Jun17_C Jun17_D Jun17_E Nov17_A Nov17_B Nov17_C Nov17_D Nov17_E
SAR11_1 SAR11_2 SAR11_3 SAR11_4 SAR11_5 SAR11_6 SAR11_7 SAR11_8 SAR11_9 SAR11_10 SAR11_11 SAR11_12 SAR11_13 SAR11_14 SAR11_15 SAR11_16 SAR11_17 SAR11_18 SAR11_19 Others
C
Marine Group II
Surface
D
DCM
MG2_1 MG2_2 MG2_3 MG2_4 MG2_5 MG2_6 MG2_7 MG2_8 MG2_9 MG2_10 MG2_11 Others
E
SAR86
Surface
F
DCM
SAR86_1 SAR86_2 SAR86_3 SAR86_4 SAR86_5 SAR86_6 SAR86_7 SAR86_8 SAR86_9 SAR86_10 SAR86_11 SAR86_12 Others
G
Actinomarinales
Surface
H
DCM
Act_1 Act_2 Act_3 Act_4 Act_5 Act_6 Act_7 Act_8 Act_9 Act_10 Act_11 Act_12 Act_13 Act_14 Act_15 Act_16 Act_17 Act_19 Act_22 Act_25 Act_26 Others

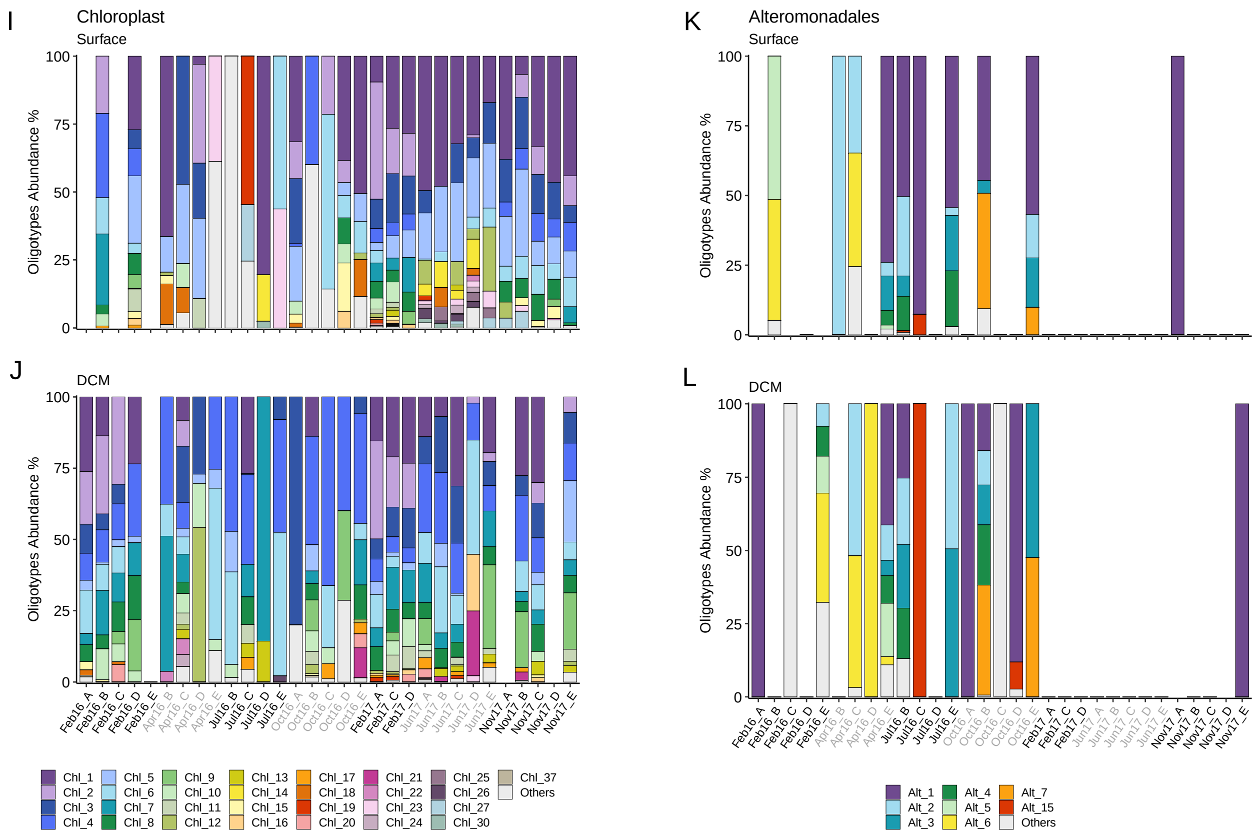

Supplementary Figure S9. Oligotypes composition of (A, B) SAR11, (C, D) Marine Group II, (E, F) SAR86, (G, H) Actinomarinales, (I, J) Chloroplast and (K, L) Alteromonadales at surface and DCM communities. Samples in the x axis are labeled according to cruise and station (Cruise_Station). Legends indicate oligotype number for each group. Oligotypes occurring in 1-3 samples are combined in ‘Others’. Missing bars indicate samples with no oligotypes found (corresponding phylotype occurring in less than 0.5% of reads). Cruise correspondence to seasons as in Fig. 1. Five samples, i.e., Feb16_A, Feb16_C and Feb16_E at surface and Nov17_A and Nov17_D at DCM, were missing. DCM: deep chlorophyll maximum.

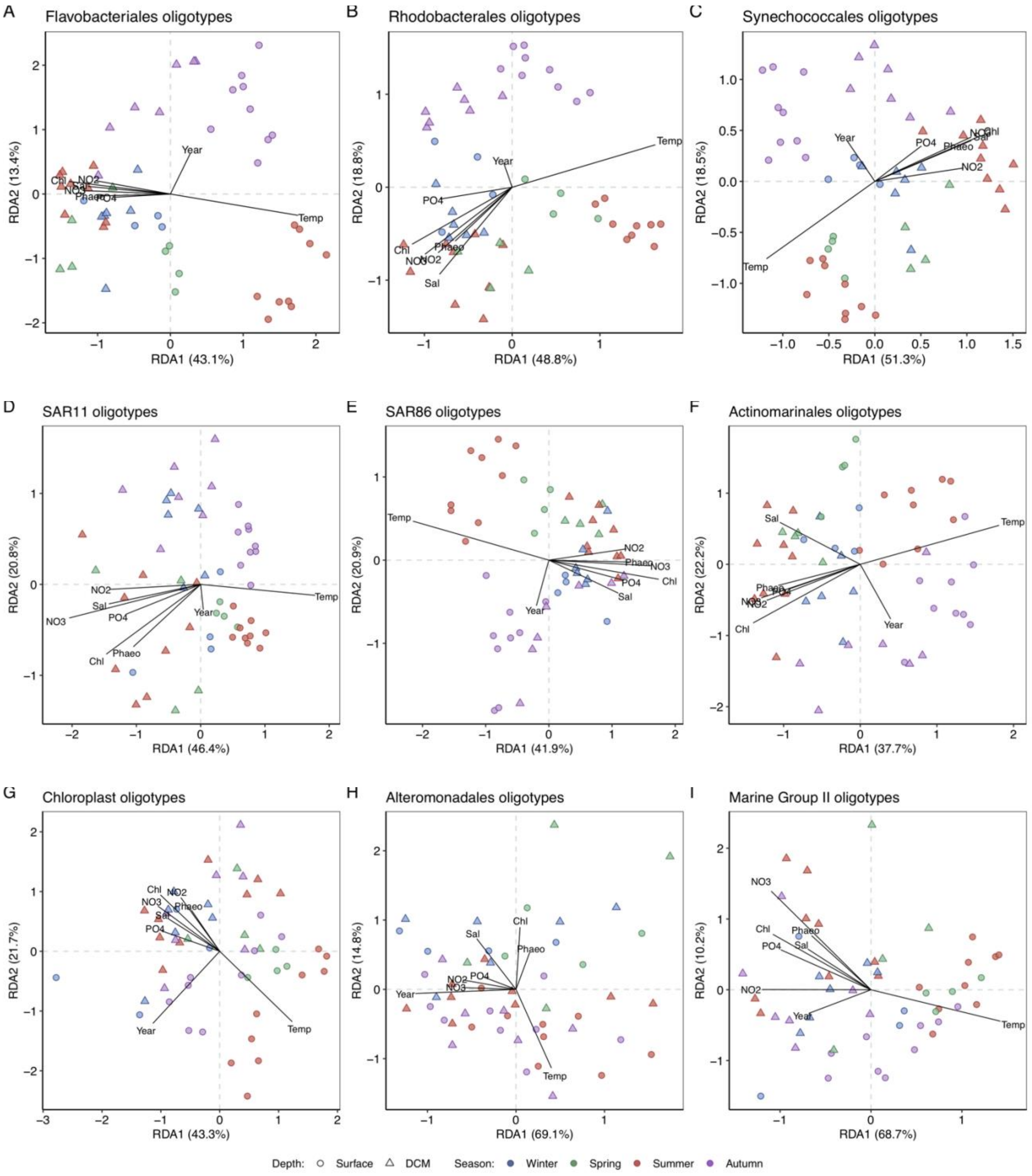

Supplementary Figure S10. Redundancy analysis (RDA) for (A) Flavobacteriales, (B) Rhodobacterales, (B) Synechococcales, (D) SAR11, (E) SAR86, (F) Actinomarinales, (G) Chloroplast, (H) Alteromonadales and (I) Marine Group II oligotypes composition with constraint variables. The percentage of variance explained is shown for each axis. Vectors represent the constrained continuous variables used in the RDA model. Strength of relative correlations with RDA axes is indicated by the length and direction of vectors. Colors indicate seasons. Temp: temperature; Sal: salinity; Chl: chlorophyll-*a*; Phaeo: phaeopigments; DCM: deep chlorophyll maximum.

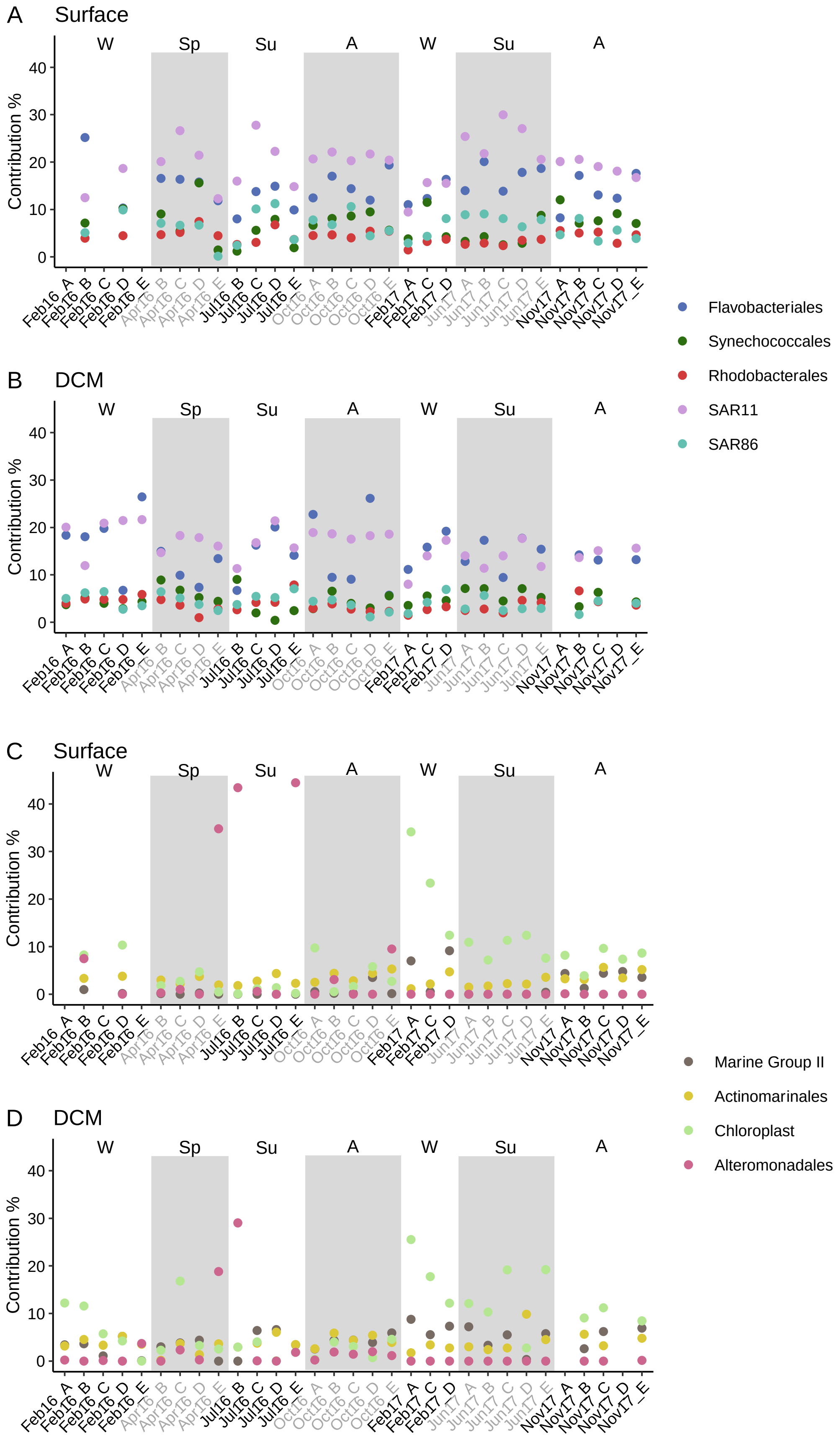
A Surface
W Sp Su A W Su A
Contribution %
B DCM
C Surface
D DCM
Feb16_A Feb16_B Feb16_C Feb16_D Feb16_E Apr16_B Apr16_C Apr16_D Apr16_E Jul16_B Jul16_C Jul16_D Jul16_E Oct16_A Oct16_B Oct16_C Oct16_D Oct16_E Feb17_A Feb17_C Feb17_D Jun17_A Jun17_B Jun17_C Jun17_D Jun17_E Nov17_A Nov17_B Nov17_C Nov17_D Nov17_E
Flavobacteriales
Synechococcales
Rhodobacterales
SAR11
SAR86
Marine Group II
Actinomarinales
Chloroplast
Alteromonadales

Supplementary Figure S11. Temporal dynamics of phylotypes relative abundances. Flavobacteriales, Synechococcales, Rhodobacterales, SAR11, SAR86 contributions to total community in (A) surface and (B) DCM. Marine Group II, Actinomarinales, Chloroplast and Alteromonadales contributions to total community in (C) surface and (D) DCM. Samples in the x axis are labeled according to cruise and station (Cruise_Station). Alternate light/shaded areas indicate changing seasons: W (winter, Sp (spring), Su (summer) and A (autumn). DCM: deep chlorophyll maximum.

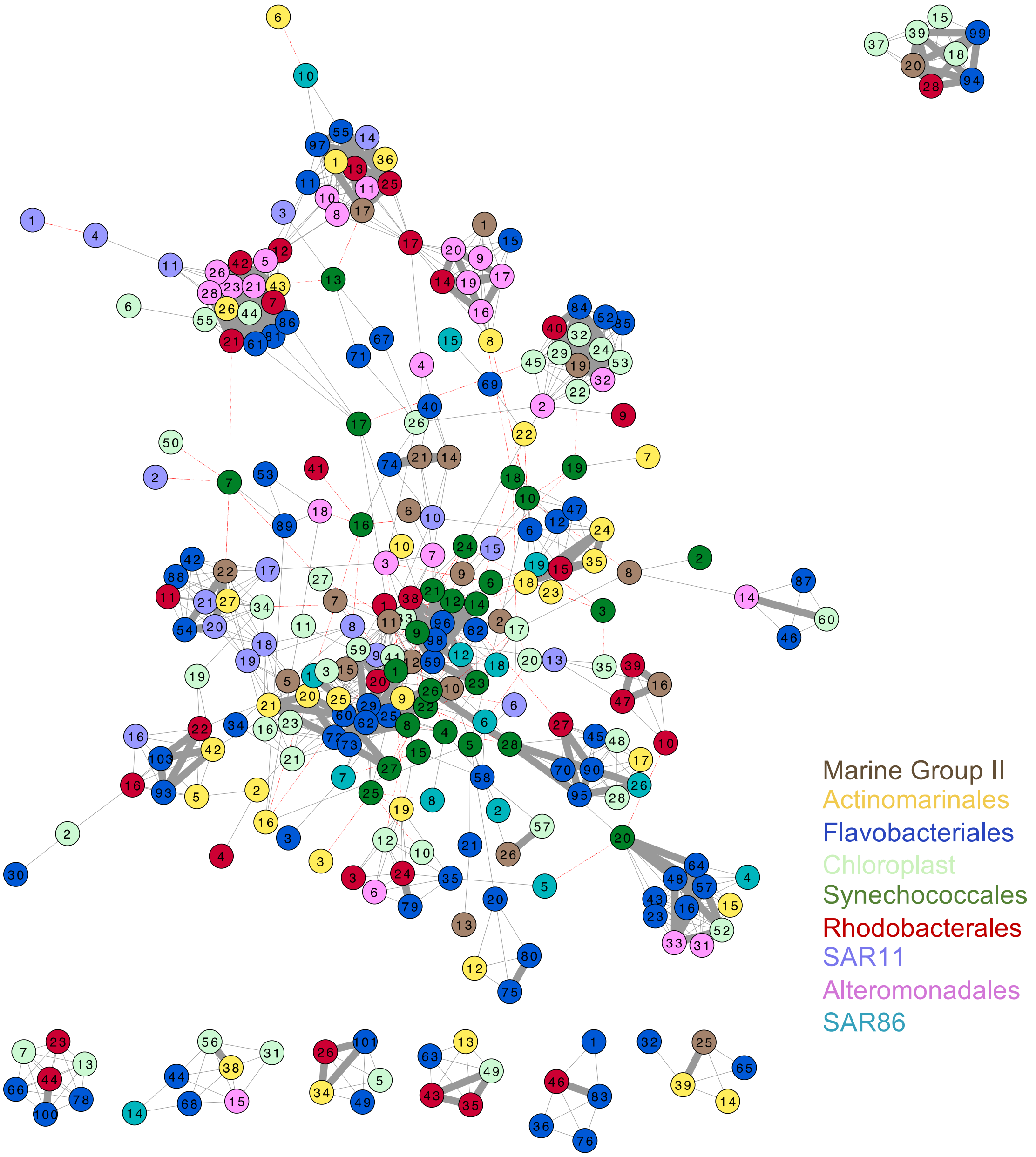

Supplementary Figure S12. Association network of oligotypes from the nine prokaryotic orders evaluated at DCM (deep chlorophyll maximum). Each node represents one oligotype, node colors indicate the nine phylotypes studied, number according to abundance of the corresponding oligotype (i.e., lower numbers indicate more abundant oligotypes). Width of lines connecting nodes is proportional to the Pearson correlation value between the two oligotypes. Solid gray lines indicate positive correlations and pointed red lines indicate negative correlations. Only significant correlations are shown (p <0.05).

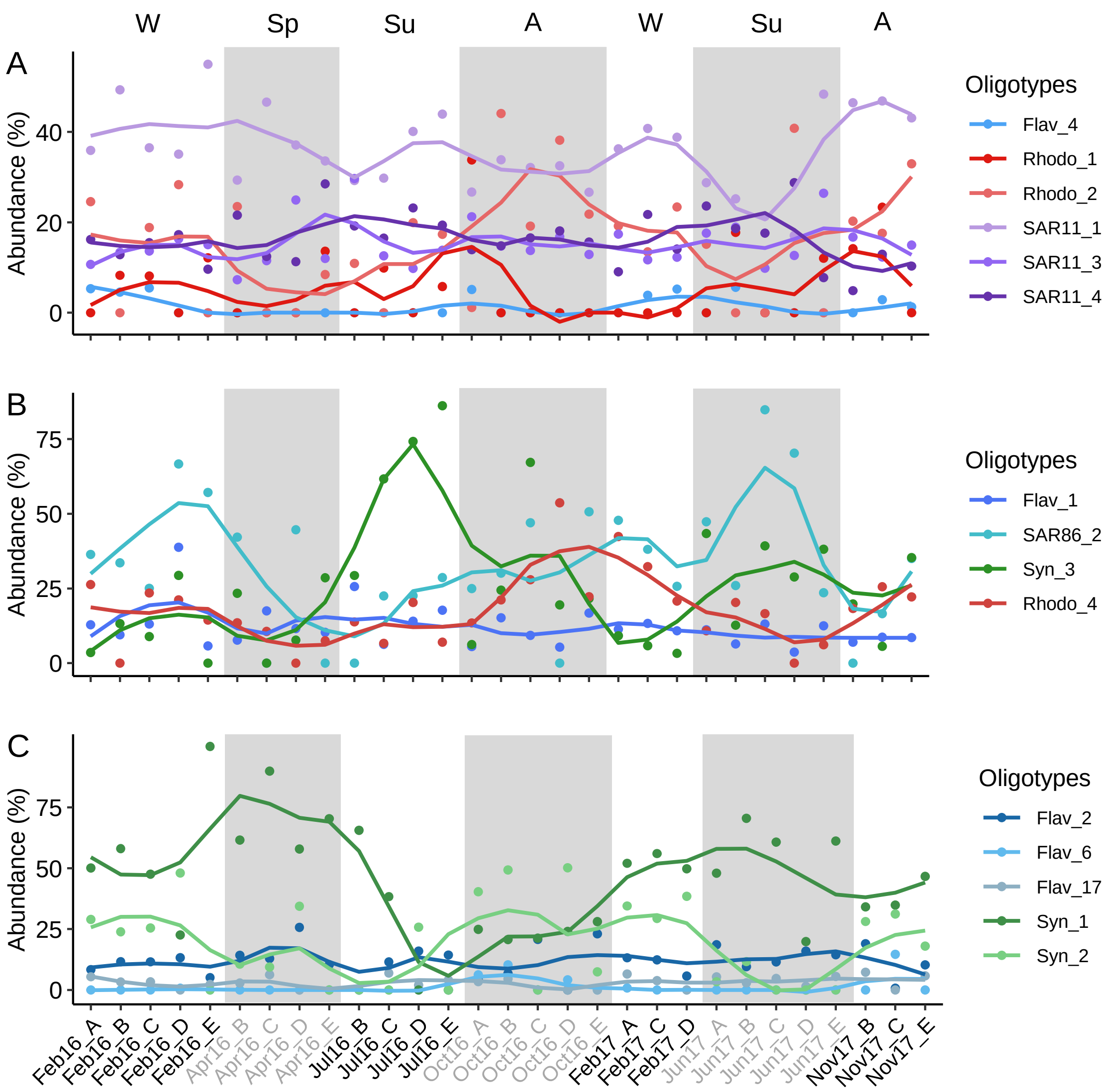

Supplementary Figure S13. Temporal variation of the three selected groups of abundant oligotypes at the DCM (deep chlorophyll maximum). The three groups of oligotypes (A, B, C) correspond to those enclosed in shaded gray areas in the surface association network depicted in Fig. 5, not correlated in DCM network. Y axis indicate the relative abundance of the oligotypes analyzed in this study. Lines are loess smoothed (smoothing function to facilitate trends visualization) and dots indicate the data value. Samples in the x axis are labeled according to cruise and station (Cruise_Station). Alternate light/shaded areas indicate changing seasons: W (winter, Sp (spring), Su (summer) and A (autumn). Oligotypes are labelled according to phylotype_oligotype number. Flav: Flavobacteriales, Rhodo: Rhodobacterales, Syn: Synechococcales.

2 Supplementary Tables

Supplementary Table S1. Abundance of total prokaryotes (PROK, $\times10^4$ cells mL^{-1}), percentage of HNA cells (HNA%), *Synechococcus* (SYN), *Prochlorococcus* (PRO) and picoeukaryotes (EUK) abundances ($\times10^3$ cells mL^{-1}). Cruise name, day (d/m/yy), station (ST) and depth for all stations are also indicated. DCM: deep chlorophyll maximum. DCM depth is indicated in parentheses.

**Cruise**	**Day**	**ST**	**Depth (m)**	**PROK ($\times10^4$)**	**HNA%**	**SYN ($\times10^3$)**	**PRO ($\times10^3$)**	**EUK ($\times10^3$)**
Feb16	6/2/16	D	0	66.14	35.96	16.81	4.73	2.50
Feb16	6/2/16	D	25	65.56	34.99	17.36	4.72	2.29
Feb16	6/2/16	D	50	62.78	34.93	15.38	6.67	2.22
Feb16	6/2/16	D	DCM (66)	43.94	45.64	2.01	5.61	2.04
Feb16	6/2/16	D	75	40.39	33.66	1.57	4.75	1.29
Feb16	6/2/16	D	100	25.65	44.30	0.33	4.97	0.31
Feb16	6/2/16	D	200	15.12	55.06	0.12	0.06	0.00
Feb16	8/2/16	C	0	47.49	34.32	14.52	3.39	2.92
Feb16	8/2/16	C	25	60.44	42.97	14.65	3.66	3.14
Feb16	8/2/16	C	DCM (30)	28.84	43.75	11.61	10.19	1.84
Feb16	8/2/16	C	75	60.68	36.86	13.86	3.75	2.88
Feb16	8/2/16	C	100	39.26	44.87	2.69	1.31	0.71
Feb16	9/2/16	A	0	48.30	41.09	13.82	3.33	3.14
Feb16	9/2/16	A	25	70.01	25.91	13.70	2.73	3.50
Feb16	9/2/16	A	DCM (33)	66.07	22.99	14.46	2.87	4.02
Feb16	9/2/16	A	50	63.50	27.49	13.48	2.94	3.38
Feb16	9/2/16	A	75	43.15	37.93	1.27	2.65	0.29
Feb16	9/2/16	A	100	40.18	42.29	1.29	1.87	0.86
Feb16	9/2/16	A	200	20.63	47.81	0.23	0.11	0.00
Feb16	19/2/16	B	0	72.66	31.78	20.35	5.76	5.21
Feb16	19/2/16	B	25	85.33	29.45	18.43	3.78	4.46
Feb16	19/2/16	B	DCM (35)	91.35	35.97	18.95	3.91	7.07
Feb16	19/2/16	B	50	87.14	32.09	15.30	1.95	4.67
Feb16	19/2/16	B	75	80.60	41.99	17.42	2.31	5.03
Feb16	19/2/16	B	100	9.39	25.65	0.36	0.26	0.03
Feb16	22/2/16	E	0	68.36	33.97	43.94	3.76	8.45

Feb16	22/2/16	E	DCM (25)	68.33	42.93	43.98	2.75	8.76
Feb16	22/2/16	E	50	38.08	59.29	10.46	7.40	2.45
Feb16	22/2/16	E	75	19.00	34.66	17.85	6.51	1.26
Feb16	22/2/16	E	100	17.71	34.59	0.07	1.11	0.08
Feb16	22/2/16	E	200	11.49	40.23	0.04	0.03	0.01
Apr16	16/4/16	D	0	63.91	27.05	31.14	0.30	2.66
Apr16	16/4/16	D	25	67.63	31.54	32.89	0.42	1.45
Apr16	16/4/16	D	50	78.85	33.13	54.08	2.66	1.08
Apr16	16/4/16	D	DCM (75)	61.84	52.54	19.98	6.30	1.60
Apr16	16/4/16	D	100	39.78	51.01	2.30	1.25	0.44
Apr16	16/4/16	D	200	28.06	56.01	0.28	0.13	0.03
Apr16	23/4/16	C	0	102.76	28.40	36.83	0.30	1.34
Apr16	23/4/16	C	25	94.88	30.40	48.76	0.47	1.94
Apr16	23/4/16	C	DCM (45)	103.05	46.66	60.52	3.77	9.26
Apr16	23/4/16	C	75	52.11	51.73	3.48	0.21	0.95
Apr16	23/4/16	C	100	47.72	47.64	2.12	0.35	0.79
Apr16	23/4/16	C	200	21.43	56.78	0.18	0.04	0.01
Apr16	26/4/16	B	0	32.77	44.75	14.39	0.32	1.37
Apr16	26/4/16	B	25	33.38	42.93	45.83	5.27	1.70
Apr16	26/4/16	B	DCM (39)	36.10	37.78	18.56	23.99	5.65
Apr16	26/4/16	B	50	16.96	29.78	3.33	2.16	1.05
Apr16	26/4/16	B	75	11.22	36.22	0.28	0.14	0.10
Apr16	26/4/16	B	100	19.40	64.96	0.28	0.08	0.12
Apr16	26/4/16	B	200	16.40	28.11	0.28	0.08	0.06
Apr16	30/4/16	E	0	37.49	46.56	13.70	0.80	1.87
Apr16	30/4/16	E	10	37.37	44.04	14.83	0.77	1.68
Apr16	30/4/16	E	25	34.53	47.63	14.19	0.71	1.38
Apr16	30/4/16	E	DCM (50)	79.82	31.12	12.97	24.82	6.64
Apr16	30/4/16	E	75	25.33	26.71	1.33	4.98	1.37
Apr16	30/4/16	E	100	19.54	30.00	0.84	0.41	0.54
Apr16	30/4/16	E	200	10.63	36.56	0.07	0.05	0.03
Jul16	11/7/16	D	0	68.44	45.59	8.51	0.22	1.22
Jul16	11/7/16	D	25	90.37	36.26	3.13	0.29	1.14
Jul16	11/7/16	D	50	97.37	43.53	10.17	1.26	1.24

Jul16	11/7/16	D	75	95.06	52.72	9.09	62.95	3.10
Jul16	11/7/16	D	DCM (81)	72.17	45.90	5.09	31.00	2.53
Jul16	11/7/16	D	100	41.48	49.92	0.81	20.48	1.06
Jul16	12/7/16	C	0	64.79	37.82	9.62	0.21	1.03
Jul16	12/7/16	C	25	67.52	35.02	3.57	0.24	1.15
Jul16	12/7/16	C	50	64.61	32.15	10.63	0.37	1.39
Jul16	12/7/16	C	75	92.93	36.92	33.74	5.91	1.06
Jul16	12/7/16	C	DCM (100)	63.41	54.16	7.81	4.89	1.74
Jul16	19/7/16	B	0	62.87	37.54	9.83	0.09	0.51
Jul16	19/7/16	B	25	86.71	36.78	7.13	0.17	1.03
Jul16	19/7/16	B	50	91.32	35.63	37.72	25.25	2.06
Jul16	19/7/16	B	DCM (58)	64.44	57.02	7.13	47.17	4.05
Jul16	19/7/16	B	75	58.35	53.47	5.39	15.17	1.70
Jul16	19/7/16	B	100	37.35	54.91	0.70	0.78	0.33
Jul16	19/7/16	B	200	20.60	60.18	0.07	0.13	0.03
Jul16	22/7/16	E	0	64.82	33.35	10.71	0.18	0.74
Jul16	22/7/16	E	10	67.04	28.37	8.24	0.14	0.71
Jul16	22/7/16	E	25	83.29	32.28	8.34	0.21	0.79
Jul16	22/7/16	E	50	77.65	44.35	25.26	3.67	1.12
Jul16	22/7/16	E	DCM (75)	58.45	54.75	2.99	19.74	1.65
Jul16	22/7/16	E	100	31.56	52.03	0.34	1.37	0.38
Jul16	22/7/16	E	200	16.74	61.51	0.09	0.07	0.01
Oct16	28/10/16	C	0	36.04	44.21	31.75	3.13	0.63
Oct16	28/10/16	C	25	40.25	46.22	36.24	3.08	0.72
Oct16	28/10/16	C	50	43.28	53.51	4.22	40.15	3.09
Oct16	28/10/16	C	DCM (55)	53.84	54.56	2.83	27.25	2.37
Oct16	28/10/16	C	75	22.04	52.06	0.31	5.89	0.30
Oct16	28/10/16	C	100	18.82	50.37	0.31	6.54	0.32
Oct16	28/10/16	C	200	18.46	36.41	0.08	0.22	0.01
Oct16	30/10/16	A	0	36.65	35.70	53.67	1.04	1.45
Oct16	30/10/16	A	10	48.19	40.10	46.99	0.43	0.87
Oct16	30/10/16	A	DCM (33)	39.83	40.18	13.80	5.67	0.50
Oct16	30/10/16	A	50	23.93	51.19	1.13	4.96	0.30
Oct16	30/10/16	A	75	16.18	51.28	0.37	4.29	0.29

Oct16	30/10/16	A	100	11.66	52.26	0.17	2.76	0.13
Oct16	30/10/16	A	200	10.34	54.61	0.07	0.11	0.02
Oct16	4/11/16	B	0	78.95	31.40	30.30	0.76	0.85
Oct16	4/11/16	B	25	101.21	32.40	50.87	8.64	1.27
Oct16	4/11/16	B	DCM (48)	68.97	48.76	10.02	27.24	1.03
Oct16	4/11/16	B	75	35.15	51.13	0.64	10.92	0.31
Oct16	4/11/16	B	100	20.42	49.72	0.15	0.09	0.01
Oct16	4/11/16	B	200	16.77	54.71	0.13	0.06	0.01
Oct16	7/11/16	E	0	78.36	39.77	25.93	1.02	0.59
Oct16	7/11/16	E	10	76.24	40.41	27.45	1.46	1.02
Oct16	7/11/16	E	25	77.16	41.61	27.83	2.02	1.03
Oct16	7/11/16	E	DCM (56)	47.35	45.11	2.59	22.31	2.15
Oct16	7/11/16	E	75	36.88	52.63	0.64	10.93	0.79
Oct16	7/11/16	E	100	28.32	57.57	0.36	3.15	0.19
Oct16	7/11/16	E	200	17.55	51.51	0.21	0.23	0.00
Oct16	11/12/16	D	0	67.14	36.30	20.42	6.43	0.89
Oct16	11/12/16	D	25	69.26	30.59	19.78	8.11	0.78
Oct16	11/12/16	D	50	72.22	35.92	14.03	9.31	0.96
Oct16	11/12/16	D	DCM (65)	58.84	38.28	6.02	6.99	0.49
Oct16	11/12/16	D	100	24.18	59.27	0.48	1.04	0.12
Oct16	11/12/16	D	200	16.29	60.66	0.07	0.18	0.01
Feb17	7/2/17	D	0	85.78	45.49	18.14	2.79	4.05
Feb17	7/2/17	D	DCM (30)	45.31	35.84	17.97	6.77	4.21
Feb17	7/2/17	D	50	68.50	41.85	5.49	5.51	1.50
Feb17	7/2/17	D	75	29.50	47.69	0.33	2.03	0.08
Feb17	7/2/17	D	100	25.02	45.06	0.11	0.16	0.01
Feb17	7/2/17	D	200	17.76	48.89	0.08	0.04	0.00
Feb17	15/2/17	A	0	58.10	44.21	8.54	5.05	7.91
Feb17	15/2/17	A	10	54.57	44.16	5.77	4.32	6.93
Feb17	15/2/17	A	DCM (20)	57.19	52.30	4.23	2.97	3.67
Feb17	15/2/17	A	50	55.40	55.36	3.37	2.81	2.62
Feb17	15/2/17	A	75	48.85	55.95	3.76	2.89	3.33
Feb17	15/2/17	A	100	45.47	58.49	3.20	2.62	2.38
Feb17	15/2/17	A	200	40.76	51.69	2.49	2.08	2.18

Feb17	17/2/17	C	0	85.01	33.91	47.42	5.86	5.67
Feb17	17/2/17	C	DCM (30)	103.03	38.11	26.31	10.66	8.39
Feb17	17/2/17	C	50	74.53	43.48	8.37	4.47	2.42
Feb17	17/2/17	C	75	80.18	48.43	10.79	5.62	3.16
Feb17	17/2/17	C	100	51.60	47.08	2.81	2.54	0.95
Feb17	17/2/17	C	200	20.33	50.43	0.11	0.04	0.01
Jun17	15/6/17	E	0	64.37	47.67	13.85	0.26	1.08
Jun17	15/6/17	E	10	70.74	42.26	7.59	0.23	1.09
Jun17	15/6/17	E	25	75.83	45.04	5.45	0.13	1.16
Jun17	15/6/17	E	50	81.72	36.13	13.09	1.18	1.63
Jun17	15/6/17	E	DCM (68)	99.43	52.05	14.04	15.57	11.57
Jun17	15/6/17	E	100	42.79	52.23	1.18	2.16	0.80
Jun17	15/6/17	E	200	23.95	48.01	0.30	0.17	0.02
Jun17	16/6/17	B	0	65.21	31.83	12.18	0.33	1.18
Jun17	16/6/17	B	25	58.17	26.78	8.97	0.16	0.91
Jun17	16/6/17	B	50	63.35	35.56	30.47	0.42	1.06
Jun17	16/6/17	B	DCM (66)	58.81	52.50	16.32	13.85	2.98
Jun17	16/6/17	B	75	40.61	52.17	1.69	5.80	0.85
Jun17	16/6/17	B	100	37.62	45.72	1.17	4.59	0.73
Jun17	16/6/17	B	200	25.34	47.32	0.23	0.10	0.02
Jun17	19/6/17	A	0	58.31	31.91	9.95	0.25	1.53
Jun17	19/6/17	A	10	59.68	35.65	9.04	0.25	1.25
Jun17	19/6/17	A	25	71.25	28.41	9.68	0.20	1.28
Jun17	19/6/17	A	50	62.27	39.12	22.13	0.39	1.42
Jun17	19/6/17	A	75	85.15	33.10	47.24	30.27	2.11
Jun17	19/6/17	A	DCM (90)	63.34	49.91	8.88	39.18	4.31
Jun17	19/6/17	A	200	22.98	50.14	0.24	0.09	0.01
Jun17	19/6/17	C	0	52.40	28.61	6.63	0.19	1.04
Jun17	19/6/17	C	25	72.01	36.20	8.04	0.22	1.12
Jun17	19/6/17	C	50	98.41	36.35	33.50	2.33	1.43
Jun17	19/6/17	C	DCM (75)	38.73	53.24	3.86	8.62	2.18
Jun17	19/6/17	C	100	24.53	55.09	0.22	0.07	0.05
Jun17	21/6/17	D	0	59.83	35.39	6.46	0.34	1.10
Jun17	21/6/17	D	25	83.46	27.78	4.66	0.16	0.94

Jun17	21/6/17	D	50	71.85	37.91	3.66	0.24	0.77
Jun17	21/6/17	D	75	77.16	38.10	10.29	18.92	0.96
Jun17	21/6/17	D	DCM (98)	53.43	49.63	1.14	41.24	2.10
Jun17	21/6/17	D	200	21.79	48.05	0.13	0.15	0.01
Nov17	20/11/17	D	0	62.87	35.68	24.73	1.88	1.08
Nov17	20/11/17	D	25	64.70	34.57	27.63	2.93	0.58
Nov17	20/11/17	D	50	59.42	34.74	28.33	3.27	0.75
Nov17	20/11/17	D	75	38.60	46.15	0.59	4.33	0.33
Nov17	20/11/17	D	100	38.18	48.56	0.55	4.64	0.33
Nov17	20/11/17	D	200	18.90	54.19	0.20	0.16	0.01
Nov17	21/11/17	C	0	75.87	48.15	28.40	5.16	2.42
Nov17	21/11/17	C	DCM (25)	73.22	52.44	21.81	5.35	2.57
Nov17	21/11/17	C	50	73.42	46.53	13.29	5.78	1.61
Nov17	21/11/17	C	75	73.30	47.71	12.49	3.99	1.48
Nov17	21/11/17	C	100	59.15	49.52	4.68	2.21	0.54
Nov17	21/11/17	C	200	22.93	52.61	0.30	1.50	0.10
Nov17	23/11/17	A	0	71.46	49.13	31.06	3.34	1.85
Nov17	23/11/17	A	10	68.66	47.37	35.42	2.94	1.34
Nov17	23/11/17	A	25	64.28	45.23	39.43	2.58	1.05
Nov17	23/11/17	A	50	66.68	32.96	36.85	2.91	0.84
Nov17	23/11/17	A	75	62.33	51.42	10.48	2.75	0.55
Nov17	23/11/17	A	100	48.75	50.59	2.78	5.71	0.72
Nov17	23/11/17	A	200	51.73	36.85	2.00	1.08	0.27
Nov17	25/11/17	B	0	73.45	28.99	32.37	1.73	1.82
Nov17	25/11/17	B	25	71.95	45.38	32.07	1.89	1.36
Nov17	25/11/17	B	DCM (50)	59.71	45.92	6.57	15.08	1.84
Nov17	25/11/17	B	75	33.20	44.52	0.41	2.97	0.24
Nov17	25/11/17	B	100	22.81	52.08	0.13	0.93	0.05
Nov17	25/11/17	B	200	15.36	47.76	0.20	0.28	0.01
Nov17	30/11/17	E	0	77.86	44.42	22.08	6.23	1.71
Nov17	30/11/17	E	10	75.02	42.07	20.89	5.92	1.39
Nov17	30/11/17	E	25	74.85	43.47	21.12	5.90	1.42
Nov17	30/11/17	E	DCM (50)	75.18	41.06	5.11	14.66	1.94
Nov17	30/11/17	E	75	40.49	37.76	0.43	4.46	0.53

Nov17	30/11/17	E	100	42.59	45.38	0.57	4.40	0.59
Nov17	30/11/17	E	200	26.06	44.23	0.47	0.53	0.05

Supplementary Table S2. Results of the distance-based redundancy analysis (db-RDA) using 999 permutations. The estimated degrees of freedom (Df), sum of squares (Sum of Sqs), F statistic value and probability (*p* value) are indicated for the model, for the first and second db-RDA axis and for the constrained variables. Asterisks indicate the significance level of each parameter (*** p <0.001, ** p <0.01, * p <0.05, no asterisk indicates p >0.05). The adjusted R^2 for the model was 0.17.

	**Df**	**Sum of Sqs**	**F**	***p* value**
**Model**	16	4.5257	1.6733	0.001***
Residual	*35*	*5.9164*		
**Axes**				
db-RDA1	1	1.3138	7.7721	0.001***
db-RDA2	1	0.5317	3.1454	0.001***
**Constrained variables**				
Temperature	1	0.6743	3.9889	0.001***
Salinity	1	0.2015	1.1821	0.147
Nitrate	1	0.3219	1.9094	0.005**
Nitrite	1	0.5006	2.9615	0.001***
Phosphate	1	0.3072	1.8173	0.007**
Chlorophyll-*a*	1	0.2023	1.1967	0.169
Phaeopigments	1	0.2100	1.2421	0.155
Station	4	0.8088	1.1962	0.054
Season	3	0.7740	1.5262	0.002**
Depth	1	0.2515	1.4876	0.032*
Year	1	0.2736	1.6188	0.024*
Residual	*35*	*5.9164*		

Supplementary Table S3. Results of the distance-based redundancy analysis (db-RDA) for surface and deep chlorophyll maximum (DCM) depths using 999 permutations. The estimated degrees of freedom (Df), sum of squares (Sum of Sqs), F statistic value and probability (*p* value) are indicated for the two models, for the first and second db-RDA axes and for the constrained variables. Asterisks indicate the significance level of each parameter (*** *p* <0.001, ** *p* <0.01, * *p* <0.05, no asterisk indicates *p* >0.05). The adjusted R_2 for the surface model was 0.12 and for the DCM model 0.12.

	Surface				***DCM***			
	**Df**	**Sum of Sqs**	**F**	***p* value**	**Df**	**Sum of Sqs**	**F**	***p* value**
**Model**	14	3.2483	1.2441	0.015*	16	2.9303	1.2213	0.001***
Residual	*11*	*2.0514*			9	1.3497		
**Axes**								
db-RDA1	1	0.7552	4.0500	0.001***	1	0.3553	2.3693	0.050*
db-RDA2	1	0.3893	2.0878	0.607	1	0.3225	2.1510	0.103
**Constrained variables**								
Temperature	1	0.4461	2.3921	0.001***	1	0.2497	1.6651	0.001***
Salinity	1	0.1806	0.9687	0.497	1	0.1866	1.2445	0.075
Nitrate	1	0.2160	1.1584	0.286	-	-	-	-
Nitrite	1	0.2888	1.5486	0.048*	1	0.1762	1.1751	0.145
Phosphate	1	0.2365	1.2683	0.164	1	0.1661	1.1081	0.225
Silicate	-	-	-	-	1	0.1947	1.2987	0.038*
Chlorophyll-*a*	-	-	-	-	1	0.2355	1.5708	0.003**
Phaeopigments	1	0.1602	0.8591	0.695	1	0.1691	1.1278	0.258
Station	4	0.6942	0.9307	0.703	4	0.5586	0.9312	0.809
Season	3	0.8012	1.4321	0.007**	3	0.6393	1.4212	0.001***
Depth	-	-	-	-	1	0.1590	1.060	0.325
Year	1	0.2243	1.203	0.162	1	0.1951	1.3014	0.045*
Residual	*11*	*2.0514*			*9*	*1.3496*		

Supplementary Table S4. Results of the redundancy analysis (RDA) for oligotypes composition of the nine groups studied using 999 permutations. Variables used for all the models were: temperature, salinity, nitrate, nitrite, phosphate, Chl-*a*, phaeopigments, station, season, depth and year. The estimated degrees of freedom (Df), variability (Var), F statistic value and probability (*p* value) are indicated for the three models, for the first and second RDA axes and for the constrained variables. Only significant variables are shown ($p < 0.05$). Asterisks indicate the significance level of each parameter (*** $p < 0.001$, ** $p < 0.01$, * $p < 0.05$). Chl-*a*: chlorophyll-*a*; phaeo: phaeopigments; temp: temperature; phosph: phosphate.

Flavobacteriales oligotypes

	**Df**	**Var**	**F**	***p* value**
**Model**	13	45.369	2.633	0.001***
Residual	*38*	*37.692*		
R_2 adj	*0.34*			
**Axes**				
RDA1	1	19.540	18.1442	0.001***
RDA2	1	6.076	5.6417	0.001***
**Constrained variables**				
Temp	1	2.938	2.7286	0.008**
Nitrate	1	2.159	2.0043	0.028*
Season	3	12.006	3.7160	0.001***
Depth	1	12.612	11.7114	0.001***
Year	1	2.360	2.1912	0.015*
Residual	*35*	*37.692*		

Rhodobacterales oligotypes

	**Df**	**Var**	**F**	***p* value**
**Model**	16	11.914	2.6288	0.001***
Residual	35	9.914		
R_2 adj	*0.34*			
**Axes**				
RDA1	1	5.818	20.5388	0.001***
RDA2	1	2.243	7.9191	0.001***
**Constrained variables**				
Temp	1	0.999	3.5264	0.007**
Phosph	1	0.590	2.0848	0.031*
Season	3	3.522	4.1448	0.001***
Depth	1	3.451	12.1825	0.001***
Residual	*35*	*9.914*		

Synechococcales oligotypes

	Df	Var	F	*p* value
**Model**	16	9.3318	3.4769	0.001***
Residual	*35*	*5.8711*		
R_2 adj	*0.44*			
**Axes**				
RDA1	1	4.7869	28.5365	0.001***
RDA2	1	1.7305	10.3164	0.001***
**Constrained variables**				
Temp	1	0.432	2.5779	0.034*
Salinity	1	0.4407	2.6273	0.023*
Phosph	1	0.4590	2.7363	0.021*
Station	4	1.2996	1.9369	0.018*
Season	3	2.5881	5.1429	0.001***
Depth	1	3.1906	19.0204	0.001***
Residual	*35*	*5.8711*		

SAR11 oligotypes

	Df	Var	F	*p* value
**Model**	16	8.0327	2.7788	0.001***
Residual	*35*	*6.3235*		
R_2 adj	*0.36*			
**Axes**				
RDA1	1	3.7319	20.6558	0.001***
RDA2	1	1.6691	9.2383	0.001***
**Constrained variables**				
Nitrate	1	0.7966	4.4092	0.001***
Nitrite	1	0.3951	2.1867	0.044*
Phosph	1	0.6381	3.5317	0.006**
Chl-*a*	1	0.5281	2.9229	0.019*
Phaeo	1	0.5057	2.7991	0.013*
Season	3	1.4882	2.7656	0.001***
Depth	1	2.2532	12.4713	0.001***
Residual	*35*	*6.3235*		

SAR86 oligotypes

	Df	Var	F	*p* value
**Model**	16	9.4343	2.0876	0.001***
Residual	*35*	*9.8860*		
R_2 adj	*0.25*			
**Axes**				
RDA1	1	3.9564	14.0070	0.001***
RDA2	1	1.9705	6.9762	0.001***
**Constrained variables**				
Temp	1	0.5989	2.1204	0.041*
Salinity	1	0.6316	2.2359	0.031*
Nitrate	1	0.5590	1.9790	0.045*
Station	4	1.6310	1.4436	0.046*
Season	3	2.1229	2.5053	0.002**
Depth	1	2.3174	8.2046	0.001***
Residual	*35*	*9.8860*		

Actinomarinales oligotypes

	Df	Var	F	*p* value
**Model**	16	13.105	1.9623	0.001***
Residual	*35*	*14.609*		
R_2 adj	*0.23*			
**Axes**				
RDA1	1	4.9425	11.8414	0.001***
RDA2	1	2.9118	6.9761	0.008**
**Constrained variables**				
Temp	1	1.1074	2.6532	0.008**
Season	3	3.6293	2.8984	0.001***
Depth	1	2.6940	6.4545	0.001***
Year	1	0.8772	2.1017	0.032*
Residual	*35*	*14.608*		

Chloroplast oligotypes

	Df	Var	F	*p* value
**Model**	16	19.810	2.3673	0.001***
Residual	*35*	*18.305*		
R_2 adj	*0.30*			
**Axes**				
RDA1	1	8.5740	16.3936	0.001***
RDA2	1	4.3041	8.2295	0.001***
**Constrained variables**				
Chl-*a*	1	1.0509	2.0094	0.046*
Season	3	4.4153	2.8396	0.001***
Depth	1	2.8244	5.4004	0.001***
Year	1	4.4153	8.4421	0.001***
Residual	*35*	*18.305*		

Alteromonadales oligotypes

	Df	Var	F	*p* value
**Model**	16	8.314	1.696	0.034*
Residual	*35*	*10.723*		
R_2 adj	*0.18*			
**Axes**				
RDA1	1	5.7460	18.7547	0.020*
RDA2	1	1.2333	4.0254	0.932
**Constrained variables**				
Station	4	3.4055	2.7789	0.007**
Year	1	1.6064	5.2433	0.001***
Residual	*35*	*10.723*		

Marine Group II oligotypes

	Df	Var	F	*p* value
**Model**	16	10.557	2.7068	0.001***
Residual	*35*	*8.5316*		
R_2 adj	*0.35*			
**Axes**				
RDA1	1	7.2508	29.7458	0.001***
RDA2	1	1.0840	4.4471	0.525
**Constrained variables**				
Temp	1	0.8507	3.4899	0.023*
Nitrite	1	0.8394	3.4434	0.019*
Phosph	1	0.6745	2.7671	0.036*
Season	3	1.7483	2.3907	0.014*
Depth	1	3.4544	14.1714	0.001***
Year	1	1.2335	5.0604	0.005**
Residual	*35*	*8.5316*		
